# Towards a health-enabling working environment - developing and testing interventions to decrease HIV and TB stigma among healthcare workers in the Free State, South Africa: study protocol for a randomised controlled trial

**DOI:** 10.1186/s13063-018-2713-5

**Published:** 2018-07-04

**Authors:** Asta Rau, Edwin Wouters, Michelle Engelbrecht, Caroline Masquillier, Kerry Uebel, Gladys Kigozi, Nina Sommerland, André Janse van Rensburg

**Affiliations:** 1 University of the Free State (UFS), Centre for Health Systems Research & Development (CHSR&D), Faculty of the Humanities, PO Box 339, Bloemfontein, 9300 South Africa; 2 Department of Sociology, University of Antwerp (AU), Research Centre for Longitudinal & Life Course Studies (CELLO), Faculty of Political and Social Sciences, Sint-Jacobstraat 2, BE-2000 Antwerp, Belgium; 3 Department of Internal Medicine, School of Medicine, Faculty of Health Sciences, University of the Free State (UFS), PO Box 339, Bloemfontein, 9300 South Africa

**Keywords:** HIV, TB, Stigma, Healthcare workers, Occupational health, Workforce, Workplace, South Africa

## Abstract

**Background:**

Occupational exposure to tuberculosis (TB) constitutes a major health risk for healthcare workers (HCWs). The HIV epidemic equally affects the workforce because of the mutually reinforcing epidemiology of HIV and TB. Stigmas associated with HIV and TB have become so intricately entangled that they stop some HCWs from seeking care in a context where serious shortages in human resources for health besiege public health facilities. It is thus imperative to research, as well as attempt to tackle, HIV and TB stigma among HCWs. But little has been done internationally—and nationally, only our own exploratory studies. Our project aims to address this by (1) scientifically assessing the extent and sources of HIV and TB-related stigma among HCWs and (2) developing and testing evidence-based, stigma-reduction interventions in public hospitals in the Free State Province of South Africa.

**Methods/design:**

The research follows a stratified cluster randomised controlled trial (RCT) design. Pre intervention, a self-administered questionnaire with the pilot study’s validated stigma scales is used to measure stigma and other key variables among randomly selected HCWs in eight hospitals—stratified by size and district and then randomly allocated to four intervention and four control sites. Interventions comprise HIV- and TB-stigma reduction activities—mainly Social and Behavioural Change Communication (SBCC) interventions—at three social-ecology levels (individual, community, and socio-structural). An outside assessor will appraise the trial mid-way through implementation. Post intervention, all baseline respondents will be followed up to complete the baseline questionnaire with additional items on interventions. Qualitative data will be collected to better understand HIV and TB stigma and explore if, and how, interventions impact stigma levels in the workplace.

**Discussion:**

The study regards as HCWs *all* staff, working in *all* different types of jobs, at *all* levels in the hospitals. Thus, the research addresses HIV and TB stigma across the whole workforce and the entire workplace. In doing so it will (1) generate essential information on stigma among HCWs and (2) implement stigma-reduction interventions that are innovative yet replicable, and potentially beneficial in addressing a pernicious human-rights-based issue.

**Trial registration:**

South African National Clinical Trials Register, registration ID: DOH-27-1115-5204. Prospectively registered on 26 August 2015.

**Electronic supplementary material:**

The online version of this article (10.1186/s13063-018-2713-5) contains supplementary material, which is available to authorized users.

## Background (Standard Protocol Items: Recommendations for Interventional Trials (SPIRIT) point 6a)

Stigma is a Greek word for a mark that was cut or burned into the skin—it identified people as criminals, slaves, or traitors to be shunned. In his seminal work Erving Goffman [1] drew on this age-old notion to define stigma as an attribute, quality, or association that significantly discredits an individual in the eyes of others. He proposed that people who do not conform to socially sanctioned norms resort to acts of concealment to protect themselves and to manage others’ impressions of them. More recent HIV literature [2–5] emphasises stigma as a *process* that involves differentiation, othering, and discrimination. Two main forms of stigma emerge: external and internal. External stigma manifests as negative attitudes and beliefs that lead people to reject, avoid, or fear those who they think have an undesirable ‘mark’ such as HIV or TB. Internal stigma refers to beliefs that a person holds about himself or herself—negative *self*-*judgements* based on that person’s lived experience, but sometimes based purely on observations of how society treats *others* with the same or similar undesirable ‘mark’. We focus on stigma because it attacks a person’s self-worth, affects a person’s right to dignity, and thus violates human rights.

We focus on HIV *and* TB stigma because of the mutually reinforcing epidemiology of the two diseases, which has led to a devastating HIV-TB co-epidemic in South Africa. HIV prevalence is high: in 2016 an estimated 18.9% South African adults aged 15–49 years were HIV-infected, with an even higher estimate of 29.7% among women in antenatal care [6]. TB in South Africa is also high, with an estimated 450,000 cases of active TB in 2013 [7].The gravity of the co-epidemic is seen in the HIV prevalence in TB incident cases in South Africa—estimated to be as high as 57% [8]. Even though the two diseases are very different—for instance, TB is airborne and curable, while HIV is predominantly sexually transmitted and incurable—people rightfully associate the two diseases because so many people with HIV die of TB. An unfortunate consequence of this is that the *stigmas* associated with the two diseases have also become intricately entangled in people’s minds and actions.

As occurs in the general population, stigma stops some HCWs from seeking care for TB and HIV [9]. Reluctance to seek help must be seen in tandem with serious shortages in human resources for health in South Africa’s public health facilities—we cannot afford absenteeism, long sick leave, or worse, attrition in our healthcare workforce. Reluctance to seek help must also be seen against a backdrop of risk. Risk of HIV infection among HCWs is likely to be similar to the general population: approximately 18.9% are HIV-infected [6]. HCWs’ risk of contracting TB is nearly four times more than the general population [10] and they may be six times more likely to be hospitalised for drug-resistant (DR) TB than the population that they care for [11]. The epidemiological realities of HIV and TB, together with HIV and TB stigma and discrimination act as ‘key barriers to both the delivery of quality health services by health providers and to their utilisation by community members and health providers themselves’ [12]. These are compelling reasons for researching HIV and TB stigma among HCWs and for developing and testing workplace interventions to reduce stigma.

Because there are so few scientific tools, particularly validated tools, to measure stigma in the public healthcare setting, it has been difficult to evaluate the success or failure of stigma-reduction interventions. This was noted by Uys et al. [9] who indicated that the majority of papers reporting stigma intervention outcomes lacked a validated instrument to measure change in stigma over time. There are several differences between our study and the limited number of others that investigate stigma in healthcare facilities. Some focus only on HIV (and not on HIV and TB) as in the case of the Nyblade et al. [12] tool for measuring stigma among health facility staff. Most focussed almost exclusively on stigmatising attitudes of HCWs towards patients and not on stigmatisation among HCWs themselves. Finally, our research spans the entire healthcare workforce and workplace, unlike other studies that measure stigma in a limited sub-set of healthcare professionals and/or workstations [13–16]. Thus, there are gaps in past and current research that indicate a clear need for reliable and validated scales to measure both HIV and TB stigma across the entire healthcare workforce and workplace.

Based on a solid theoretical framework, and our findings from a series of small preliminary studies [17, 18], the current proposal team Research Centre for Longitudinal & Life Course Studies-Centre for Health Systems Research & Development (CHSR&D-CELLO) worked closely together in 2012–2013 to develop and refine a range of scales to measure the most important dimensions of TB and HIV stigma: external and internal stigma [19, 20]. External stigma is directed by HCWs *outwards* towards other HCWs, and internal stigma is directed by HCWs *inwards* towards themselves. The resulting parallel scales—measuring external and internal stigma towards HIV *and* TB—were piloted in Pelonomi Regional Hospital (Free State, South Africa; Stigma Score Pilot Study; ethical clearance: ECUFS NR 192/2012). Subsequently the pilot stigma scales were validated and the results published in *Clinical Infectious Diseases* [21].

At the same time that the Stigma Score Pilot Study was being conducted, insights from an academic literature review on stigma-reduction interventions, particularly in healthcare settings, were used to inform workshops with key stakeholders, out of which a series of stigma-reduction interventions designed to work at different socio-ecological levels (individual, community, and structural) were proposed and discussed. The work of Li et al. on HIV stigma among healthcare workers (HCWs) in China [22–24] strongly influenced our decision to apply the theory of the Diffusion of Innovations [25] to intervention design. The theory recommends the deployment of change agents—or as Li et al. call them, popular opinion leaders—to promote socio-behavioural change. Results of their trial involving 1760 service providers in 40 hospitals in China found that hospitals delivering HIV-related stigma-reduction interventions reported more desirable intervention outcomes than those in control hospitals [26]. Raising awareness of HIV and TB stigma is quite common in wider societal- and community-level contexts in South Africa. Among the myriad and mixed results of such efforts are indications that communication interventions can have an adverse effect, and *increase* stigma [27]. Nonetheless, it seems more often the case that stigma-reduction campaigns across different media and communication platforms are beneficial, at least to some degree (cf. [28, 29]). The Social and Behavioural Change Communication (SBCC) intervention design we finally decided on combines the Diffusion of Innovations change agent/popular opinion leader approach with a social marketing campaign that promotes a locally relevant, culturally sensitive slogan and image targeting all HCWs with a single stigma-reduction message. The initial research on interventions and our piloted imagery were subsequently published in *Global Public Health* [30].

To the best of our knowledge—as informed by a comprehensive review of available academic literature—ours are the first research studies conducted on HIV and TB stigma among HCWs in South African healthcare facilities. Without having first established the extent and sources of HIV and TB stigma in the South African healthcare workforce, it would be premature to conduct research that compares this phenomenon in South Africa with what occurs in other countries or regions. Therefore, for our current randomised controlled trial (RCT) we chose as comparators eight randomly selected public hospitals (four intervention vs four control) situated in the same province of South Africa—the Free State (SPIRIT point 6b). This choice is sound in terms of the number of possible comparators (27 hospitals) as well as in terms of geography and expertise. The *Centre for Health Systems Research & Development (CHSR&D)* is situated at the University of the Free State (UFS) in Bloemfontein, in the middle of the province. The CHSR&D was established 25 years ago in the Faculty of the Humanities and has considerable expertise, as well as working partnerships, built over decades of research in the province’s public health facilities. The University of Antwerp (AU), Faculty of Political and Social Sciences, Department of Sociology, *Research Centre for Longitudinal & Life Course Studies (CELLO)* has a very long history of fruitful research partnership with the CHSR&D and also has excellent working knowledge of public health in the province.

In response to the research needs outlined above, as well as to extend the preliminary work of our *Stigma Score Pilot Study*, the CHSR&D and CELLO designed a randomised control trial with two key objectives (SPIRIT point 7) in mind: (1) to measure stigma by scientifically assessing the extent and sources of HIV and TB stigmatisation among the healthcare workforce and (2) to design, refine and test evidence-based, stigma-reduction interventions in randomly selected public hospitals in the Free State Province of South Africa.

This article presents the protocol for the RCT, and is written to comply with the recommended SPIRIT guidelines for RCT protocols [31] (Additional file 1).

## Methods/design

### Trial design (SPIRIT point 8)

The research follows a stratified cluster RCT design. Pre intervention, a self-administered questionnaire with the pilot study’s validated stigma scales was used to measure stigma and other key variables among randomly selected HCWs in all eight randomly selected sites. The intervention comprises of stigma reduction activities at three social-ecology levels (individual, community, and structural) in the four intervention sites. To evaluate implementation, an outside assessor will appraise the trial mid-way through its implementation, partly by collecting qualitative data from purposively selected HCWs. Post intervention, all respondents from baseline will be followed up using the same scales with added questions on interventions. Quantitative and qualitative data will be collected to assess the size of the impact as well as to uncover the processes through which the interventions may impact on stigmatisation.

### Study setting and site selection (SPIRIT point 9)

The study settings are public hospitals in the Free State Province, South Africa. From the total population of 28 hospitals that could be selected as comparators, one was eliminated (Pelonomi Regional Hospital) because it was the site of the Stigma Score Pilot Study.

In order to ensure an equal distribution of large and small hospitals across the different districts in both intervention and control arms, hospitals of similar size (based on numbers of staff), and where possible in the same district, were deliberately paired. Within these pairs, a coin toss was used to randomly allocate hospitals to Arm A or Arm B, and then again to allocate the two arms to intervention or control. The number of hospitals required was estimated considering the fact that the number of staff members across the hospitals in each group differ. The average number of staff members per hospital considered is 220. The same parameters were used as those used to calculate the sample (described later), and the number of hospitals required was estimated to be approximately four hospitals per group (intervention / control). A full list of the sites appears in the addenda (Additional file 2).

### Participants

The study regards as HCWs *all* staff, working in *all* different types of jobs, at *all* levels in the hospitals. Thus, we are measuring stigma and intervening across the whole workforce and the entire workplace.

#### Sampling (SPIRIT point 14)

The parameters used for sample size calculation were estimated from the Stigma Score Pilot Study. The mean stigma score considered for the control group was 0.85 with a standard deviation of 0.71. The stigma score is expected to be reduced by approximately 24% to 0.65 in the intervention group with a standard deviation of 0.39. The two-tailed test is considered with Type I error of 0.05 and Type II error of 0.10, to give 90% power. The coefficient of variation considered for each group is 0.25 and the intra-cluster correlation coefficient is 0.05. The estimated sample size per arm is 173 and the estimated inflation factor (design effect) is 1.7. Therefore, the required sample size for this study per arm is approximately 173 × 1.7 ≈ 292 participants. The total number of participants required for this study is 584 (347 respondents in intervention sites and 237 in control sites).

The same HCWs participate in baseline and the post-intervention surveys. Based on our extensive experience of fieldwork in Free State hospitals [32], we estimate that the baseline survey will need 50% oversampling of respondents to allow for loss-to-follow up between baseline and post-intervention surveys (SPIRIT point 15). In practice, a random sample of 882 HCWs was drawn from three broad job categories: *healthcare professionals* (446) (physicians, nurses, and allied HCWs such as psychologists, pharmacists, etc.); *management and administrative staff* (116); and *support staff* (318) (e.g. messengers, cleaners, porters, housekeeping, catering, security, etc.). Two respondents did not indicate their job category.

For the baseline and follow-up survey components of the study a sampling frame (SPIRIT point 16a) for individual respondents (and replacements) was drawn up for each hospital site using its most recent, up-to-date database of employees. Employees were allocated to the following three occupational groups: (1) clinical professionals (including physicians, nurses, and allied healthcare professionals such as physiotherapists, occupational therapists, speech therapists, dieticians, pharmacists, social workers, radiographers, etc.); (2) management and administrative staff (e.g. chief executive officer (CEO), finance officers, clerks, etc.) and (3) support staff (e.g. household aids, messengers, porters, etc.). We sampled participants from each occupational group proportionally to the overall size of the groups *in each hospital*. Random sampling was used to select participants for inclusion into the study. For each occupational group, this entailed arranging names alphabetically and using Excel 2010 to randomly assign numbers to each staff name. The random numbers and corresponding staff names were then arranged in ascending order before selecting the required number of staff within each occupational group. All remaining staff on the list were considered as replacements in the case of refusals and staff not being available. Replacement staff names were selected in ascending order of appearance of the random numbers. In total, 24.0% of HCWs were not able to participate (no longer working there, unavailable, refusal) and had to be replaced.

#### Recruitment (SPIRIT point 15; see also Section 3.3.1)

Over its long history of working in Free State public health facilities, the CHSR&D has developed effective mechanisms to recruit participants. We begin by consulting closely with provincial managers and then proceed to consult with individual hospital CEOs; these communications aim to make sure that the study and its details are properly communicated to the relevant top managers. The CEOs then generally call a meeting of other managers operating at all levels throughout the facility. At this crucial meeting we present the research and explain what is required to support it, including the number and distribution of staff required to participate. They then advise on best recruitment processes. Recruitment always requires research fieldworkers—as well as implementers—to be very flexible and sometimes work after hours in order to accommodate staff on different shifts, and allow for the relative time and workload pressures experienced in different jobs and in different types of wards.

Fieldworkers are very well trained and sensitised to ethical protocols and practices. Each one is given a list of the randomly selected participants who he/she needs to locate, approach with introductory information about the research, and invite to participate. Finding selected candidates usually entails working through ward managers or matrons, line managers and supervisors. People who are willing are then taken through the full informed consent process by the fieldworker, and can opt to do this in one of three local languages (English, Sesotho, or Afrikaans). If the fieldworker is not proficient in the person’s language of choice, he/she will hand the participant over to a fieldworker who is. Fieldworkers can easily contact one another or the on-site fieldwork manager/s, as they all carry cell phones with airtime.

Fieldworkers attempt to contact persons on their list at least three to four times. Where the potential participant is on leave or off sick for the duration of the data collection in that hospital, or if they do not agree to participate, the fieldworkers proceed systematically through their list of randomly selected replacement candidates. These processes are repeated until the required sample of participants for the surveys is obtained.

Although the *processes* described in this section are similar for recruiting survey respondents and intervention participants, the latter are not sampled in the same way. Selection for interventions is described in the next section.

## Interventions (SPIRIT point 11a)

It is accepted—particularly in prevention science—that a combination of interventions is more effective at bringing about desired change than a single intervention [33–35]. Thus, a mix of interventions— *clinical*, *structural*, and the most important, *socio-behavioural*—is proposed for simultaneous rollout. Interventions are informed by insights from key publications [24, 36–39]. Stigma-reduction activities also target three communication levels—*individual, community, and socio-structural*.

As indicated in the ‘Background’ section, interventions are based on a comprehensive review of the academic literature—particularly literature on stigma as it relates to HCWs—including our own preliminary studies on issues that impact stigma in FSDoH workplaces [17, 18, 30], and piloting of social marketing materials [30].

### The clinical intervention

This is being implemented by Dr. Kerry Uebel—a physician with the Free State Department of Health (FSDoH) and a member of the research team. She will support occupational health (OH) nurses—and, where OH units do not exist, staff physicians and nurses who test and treat facility staff for HIV and TB—to provide screening and treatment for TB and human immunodeficiency virus (HIV)/acquired immune-deficiency syndrome (AIDS). This is already part of her work, so the project ‘piggy backs’ on her efforts in this regard. What is extra is that she will also assist with any issues and questions that arise from the stigma-reduction workshops for HCW change agents (described below).

Because we might not get physicians to attend the workshops, and because as a physician she is perfectly positioned to be heard by her professional peers, she will promote awareness of HIV and TB stigma among physician in intervention sites via a PowerPoint presentation at one of their monthly clinician’s meetings.

### Structural interventions

These are embedded in clinical and socio-behavioural communication components. The clinical knowledge and expertise of HCWs, as well as their knowledge of HIV and TB stigma should be strengthened by having a clinician assist them with their cases. A positive outcome we anticipate from this is that health systems will be strengthened by an improved environment in which HCWs can seek care in the workplace.

Knowledge of key infection control practices, and distribution of information on HIV and TB stigma-related rights and responsibilities in the healthcare workplace, including where to go for help and how to report transgressions of HCW rights—are all examples of structural issues embedded in training sessions.

### Social and Behavioural Change Communication (SBCC) interventions

These are the priority interventions and comprise two components: training HCW change agents, and a social marketing campaign.

#### The main SBCC intervention is training HCWs to reduce HIV and TB stigma in their workplace

This is underpinned by social theory of the Diffusion of Innovations [25] proposed by Everett Rogers. The theory sets out to explain how, why, and at what rate, new ideas and technologies spread. He proposes that social change is accelerated via change agents: individuals who are seen as role models. People look up to them and regard their choices and actions as admirable and worthwhile emulating. As a consequence of this esteem, they are ideally positioned to promote change and the diffusion of new ideas and practices in social and organisational contexts [40]. This social theory of Diffusion of Innovations has been applied to stigma among HCWs in other settings [22–24].

The research design regards *all* HCWs as contributing to, and thus co-constructing, their workplace community and its social norms. Accordingly, change agents need to be identified across the entire workplace, and from all three occupational categories: clinical staff (e.g. physicians, nurses, allied health workers); managerial and administrative staff; and support-services staff (e.g. cleaners, laundry workers, housekeeping, porters) (SPIRIT point 10; see also ‘Section 3.4’). Adopting multiple methods for selecting change agents helps to identify ‘informal’ as well as ‘formal’ leaders and yields candidates across a wide social spectrum; thus, a two-pronged sampling approach—*positional* and *snowball*—will be followed to identify and recruit them [41].

Positional sampling involves selecting individuals based on the formal leadership positions that they hold in their hospitals. An advantage is they are well-positioned to promote change. A drawback is that only those in formal leadership positions are accessed. To remedy this Valente and Pumpuang [41] suggest combining positional with snowball sampling. In snowball sampling the pool of change agents serves as a sociometric sample that provides information for locating other potential change agents.

The first set of individuals—the ‘index cases’— are identified via positional sampling. We ask hospital CEOs to provide a list of their most influential staff across all three staff categories, e.g. top and middle managers (including the CEOs themselves), members of the hospital committees, occupational health nurses, health and safety representatives, social workers, and so forth. We then hold an information meeting for these positional leaders and invite them to act as change agents. In the first iteration of snowball sampling, they are also asked to nominate other staff who they think are role models in their facility and who they think are likely to be effective in helping to reduce stigma; nomination can be vertical or horizontal in terms of levels of hierarchy and occupational categories. These new nominees are then approached by fieldworkers and the intervention team leader, invited to be change agents, and also asked to nominate others. This snowballing process is repeated until the required number of change agents is recruited. *As usual, informed consent must be obtained from all who agree to participate (SPIRIT point 32)* (Additional file 3). In terms of process, the names of consenting nominees and other relevant information are entered into an Excel spread sheet by the research and implementation teams (SPIRIT point 16a). All consenting nominees appearing in the Excel sheet are then invited by team members to participate in stigma-reduction training at selected dates (SPIRIT point 16c).

It is estimated that a sample of 40–50% from each staff category—clinical, management and administration, and support services—is needed to effect measureable change in their hospitals (SPIRIT point 14). Half of the required proportion of participants were contacted for the first round of training in all four intervention hospitals; the second half will be contacted in September 2017 for the second round of training. We split the training into two rounds because we did not want any one hospital to lag behind too much in terms of obtaining the first full ‘dose’ of the intervention; this is more equitable in terms of time, which is important given that we do not have as much intervention time as we initially anticipated (see the ‘Discussion’ section). The estimated number of change agents for training in each intervention hospital appears in the addenda (Additional file 4). For the first round of training the required sample size was reached after the second round of peer nominations (snowball sampling). It is anticipated that the same will occur for the second round of training (SPIRIT point 15; see also ‘Section 2.3.2’).

It is important to note that, in order to minimise disruption of service delivery, training times are arranged in consultation with hospital representatives/management.

Training sessions are standardised as much as possible. Facilitators practice together and deliver the training strictly adhering to a facilitator’s training manual [42], reviewed by peers, and translated (as well as back-translated) into the three local languages. To minimise impact on the workplace, a training session lasts only 4 h. On completion of the training participants evaluate the training and receive a certificate of attendance. Training follows best practice in terms of content and processes that are highly interactive and lively, designed for adult-based learning and for engaging participants at the levels of knowledge, emotion, and action.

At first we planned to train participants in separate groups depending on their job categories. But, as our pilot training sessions showed, this is not possible because one cannot take too many clinical professionals, for instance, away from their posts at one time. We were also advised that splitting sessions into people from one job category could be stigmatising in itself, and constrain the abilities of people at different levels in the hierarchy to communicate and learn from one another about an issue which transcends job categories. We thus divided trainees into groups in terms of one main criterion: their preferred language of instruction (SPIRIT point 16a). Trainees who did not have a particular language preference were allocated to training sessions according to their expressed preference for certain dates and times.

What is unique to the training is that it builds on ordinary human compassion, expressed in caring *communication*. So, there is no monetary or infrastructure costs. Change agents are encouraged to start conversations about HIV and TB stigma in the workplace with co-workers, and/or respond to stigmatising situations they encounter in the workplace, in ways that feel *natural* to them. They do receive some social marketing materials (e.g. branded wristbands and pens) to support their stigma-reduction communications, and there is also the wider, visible social marketing campaign (especially the posters) that they can point to in order to spark conversations or highlight what they are saying.

#### The second SBCC intervention is a social marketing campaign promoting stigma reduction

Targeting all levels of staff, and all spaces in the hospital, the social marketing campaign is designed around one branded slogan and one branded image. The slogan (in three languages) says: *Let us Stop stigma. Be kind to yourself. Be kind to others*. This refers back to theory and the division of stigma into *internal* stigma (be kind to yourself) and *external* stigma (be kind to others). The image is very bold: a large open hand, positioned like a stop signal, in a red *shwe-shwe* design. *Shwe-shwe* is a traditional South African printed fabric much loved by people of all races and from all walks of life.

The slogan is repeated on different sized posters displayed throughout the different hospital wards, as well as administrative and meeting spaces. There is also a range of promotional gifts (pens; chocolates, wristbands; fridge magnets) branded with the same slogan and image, which we hand out to everyone in the facility.

Social marketing materials can be very short-lived in a recipient’s memory. This is why we have the same message and the same image communicated in different media and in different spaces, and why we hand out promotional gifts throughout the hospital on more than one occasion.

### Eligibility criteria for the interventions (SPIRIT point 10; see also ‘Section 3.3.1’)

To summarise, for the *clinical* intervention participants must either be occupational health unit staff, and, where occupation health units (OHUs), do not exist, physicians and nurses who test and treat facility staff for HIV and TB. Eligibility criteria for *attending the training workshops* are that participants who are recognised and nominated as change agents or potential change agents are selected by facility staff at all levels (not researchers) via two processes: positional sampling and snowball sampling.

Eligibility criteria for *facilitating the workshops* is that facilitators should be experienced and accredited by SAQA (South African Qualifications Authority). The facilitation team comprises of three such professionals, especially recruited, who, between them, can deliver the training in all three local languages.

### Criteria for discontinuing or modifying interventions, and for monitoring HCW actions

Participation in the interventions, as for the surveys, is voluntary and by consent, so participants can withdraw at any time without fear of prejudice (SPIRIT point 11b), and without fear of having their names reported in the workplace by the research or implementation teams.

In terms of the communication actions of HCW change agents, spontaneous and private conversations cannot be monitored or measured (SPIRIT point 11c). What we do is research their experiences, discussed later in the ‘Data collection’ section.

It is not anticipated that interventions will change at this point in time because they have already been extensively workshopped in collaborative meetings with all key FSDoH stakeholders, they have been piloted, the first in two series of training sessions has already been implemented in each of the four intervention hospitals (between June and July 2017), and the first set of social marketing materials has been distributed. The second series of training sessions is anticipated to take place between September and October 2017.

### Participant timeline (SPIRIT point 13)

**Fig. 1:**
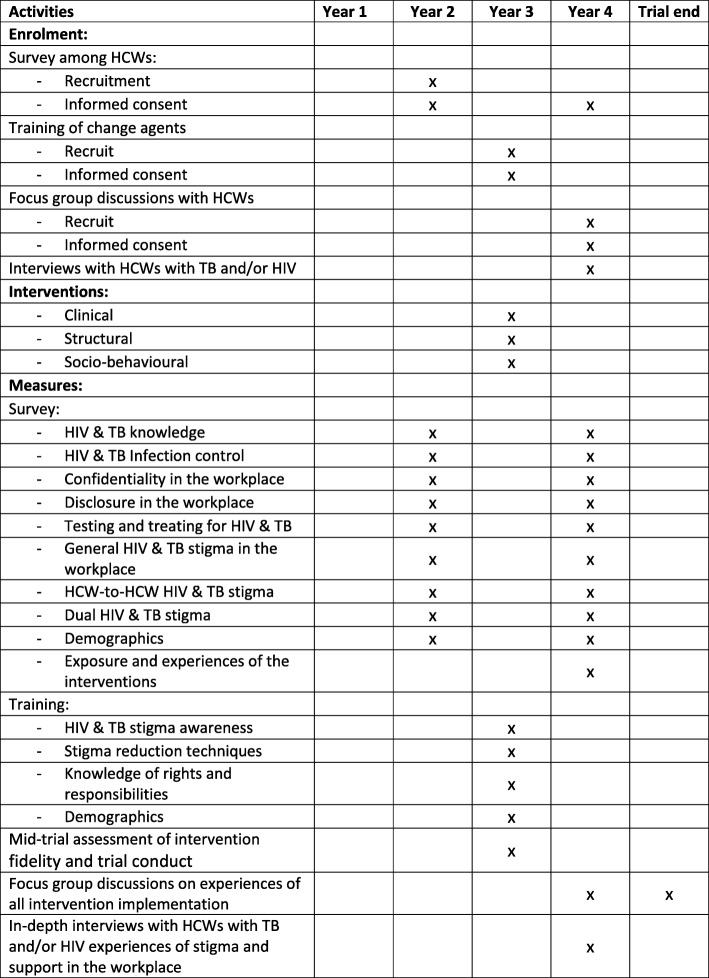
Schedule of enrolment, interventions, and assessments. Template Source: Chan et al. [31]

Figure 1 SPIRIT Table [31] shows details on the schedule of enrolment, interventions, and assessments.

## Outcomes (SPIRIT point 12)

Primary outcomes:

1. Measurement of the extent of HIV- and TB-related stigma among the public healthcare workforce in the Free State Province of South Africa, and the generation of information on the sources of such stigma.
  (Time points: rough estimates calculated by December 2016 to inform partners’ decision on trial continuation; Year 4, December 2018, calculate the change in the extent of stigma by comparing measures obtained in baseline statistics *with* measures generated in post-intervention survey data)
2. Evidence-based, stigma-reduction interventions are developed and tested in randomly selected public hospitals in the Free State Province of South Africa.
  (Time points: start of interventions in Year 3, March 2017 to Year 4, December 2018 when quantitative data from the post-intervention survey, and the qualitative data from focus groups and interviews are generated and analysed)

Secondary outcome:

The environment in which HCWs in intervention hospitals seek care is improved.

By ‘environment’ we mean, for instance, that greater awareness of HIV and TB stigma—and hopefully diminished stigma—can create a workplace that is more sensitised and empathetically responsive to human rights. We also anticipate that when staff who test and treat other staff within a facility are well supported clinically, they are likely to provide more confident and accurately tailored services. In turn, this could encourage HCWs to seek and use health services provided in the workplace, which diminishes time taken off work and should help to some extent to alleviate HCW shortages and associated pressures on other HCWs.

(Time point: Year 4, December2018, when quantitative data from the post-intervention survey, and qualitative data from focus groups and interviews are generated and analysed)

## Data collection and management (SPIRIT point 18a)

Fieldworkers experienced in health research are used for all data collection stages and given additional training to sensitise them to the requisite quality and standardisation of research processes. In the case of qualitative work, fieldworkers work side-by-side with CHSR&D research team members.

Small tokens of appreciation in the form of a shopping voucher (for R50; approximately US$4) and small gifts (chocolates, pens, and wristbands branded with the imagery and slogan of the social marketing campaign) are supplied to fieldworkers and researchers to give to participants who complete the surveys, participate in focus groups/ one-on-one interviews, or attend the change-agent training.

### Baseline data collection

Once the fieldworkers track down the randomly selected individuals and obtain informed consent from those willing to participate (described earlier in the section ‘Participants’ sub-section ‘Recruitment’) (*SPIRIT point 32*), (Additional file 3), they hand each participant an envelope with the stigma questionnaire—to be self-administered. Fieldworkers either wait for a respondent to fill in the questionnaire, or arrange a time and place to collect the completed questionnaire from the respondent if it is not convenient for the respondent to fill it in immediately. Respondents with low levels of reading literacy are gathered into small groups, the fieldworkers work through the questions verbally *with the whole group*, and address any queries with the whole group; however, respondents have to fill in the questionnaires themselves.

When the respondent hands in their completed questionnaire, the fieldworker quickly scans through each one to check for completion (not to see the person’s actual answers). Where substantial sections of the questionnaire are not completed, the fieldworker marks the places and hands it back to the respondent to complete properly. This helps to ensure that the dataset is not overly compromised by being incomplete.

As described in the ‘Ethics’ section, respondents’ names are replaced with a unique number that is pasted onto their consent forms, their completed survey questionnaires, and the participant list kept by the fieldworkers. Each day, the lists, consent forms, and completed questionnaires are handed to fieldwork team leaders, who are responsible for collating information at the end of each day (i.e. how many respondents are reached, number of questionnaires completed/outstanding, and highlighting which respondents still need to be reached). The fieldwork teams maintain telephone contact with the CHSR&D on a daily basis and once a week they meet face-to-face with one of the project principal investigators (PIs). The purpose of this meeting is to report back on progress, any difficulties experienced, and hand in completed questionnaires.

The baseline questionnaires included the stigma scales from the Stigma Score Pilot Study (HIV stigma among HCWs; TB stigma among HCWs); the validation of these scales was published in *Clinical Infectious Diseases* [21]. Three new scales were added (General climate of *HIV* stigma in the hospital; General climate of *TB* stigma in the hospital; HIV and TB stigma combined). The baseline stigma scales are provided in the appendices (Additional file 5); (SPIRIT point 18a).

There were also several other sections collecting data on important stigma-related issues (demographics; basic and advanced HIV and TB knowledge; HIV and TB infection control in the workplace; confidentiality in the workplace; HIV status disclosure in the workplace; testing and treating for HIV and TB; and knowing people with HIV and TB). The full baseline instrument is available from the corresponding author on reasonable request.

### Post-intervention data collection

The same processes and protocols are followed for the post-intervention survey. Respondents from the baseline survey are tracked down via their unique identifiers and asked to complete the post-intervention questionnaire. As noted before, there was a 50% oversampling of respondents to allow for loss-to-follow up between baseline and post-intervention surveys (SPIRIT point 18b).

To ensure comparability, the post-intervention survey will resemble the pre-intervention survey, thus including the HIV/AIDS and TB stigma scales as well as the range of other key outcome measures related to stigma at the individual, community, and structural levels—as described above. The post-intervention survey will also have a new section to measure the reach and appeal of the stigma interventions.

### Qualitative data collection

Qualitative data are collected at several junctures during the trial. Data collection always complies with informed consent and protocols discussed later, in the ‘Ethics’ section.

As agreed in the Memorandum of Agreement (MoU) between FSDoH and CHSR&D, mid-way through the interventions an outside assessor recruited by the FSDoH collects data on the trial interventions and trial conduct from purposively selected individuals.

Following the change-agent training sessions, approximately 10 focus group discussions comprising 8–10 people each are held with purposively selected HCW change agents who attended the training. All sessions will be audio-recorded. The aim of is to see *if* and *how* the change agents mobilised the knowledge of HIV and TB stigma that they gained from the training workshops and how, if at all, they were able to use some of the stigma-reduction communication techniques they learned.

Audio-recorded focus group discussions may also be held with purposively selected HCWs at all levels who did *not* attend the change-agent training. The broad aim is to explore their perceptions of HIV and TB stigma and if and how it manifests in the workplace. It will also be interesting to probe their awareness and reception of the social marketing campaign and its effects.

Qualitative data are also collected by CHSR&D researcher team members via one-on-one in-depth interviews, audio-recorded, with any HCWs who are/were infected with HIV and/or TB and who are willing to share their experiences of stigma and support in the workplace.

### Data processing and management (SPIRIT point 19)

#### Baseline data processing and management

The CHSR&D has a dedicated, secure data processing room, as well as a dedicated secure data storage room. All UFS computers are password protected; in addition project data files are encrypted.

A quarter of the collected questionnaires are randomly selected for purposes of developing a coding list for open-ended questions. Four data coders collaboratively develop the coding list. To ensure quality and accuracy, data are double-captured and cleaned in SPSS. Frequency counts are calculated for all questions to check for outliers. The two datasets are then checked for any discrepancies and corrected.

As noted in the ‘Ethics’ section below, no respondent names appear in the SPSS database, only their unique identifying numbers. The list with the names of participants will be kept separate from the database, and will only be used for the post-intervention survey, when we need to know who to follow-up. All research data and documents referring to the trial will be stored and maintained in a secured storage space at the CHSR&D, UFS, for a minimum of 10 years from the end of the trial.

#### Post-intervention data processing and management

The same processes and protocols are followed for the data gathered from HCWs’ evaluation of training interventions, as well as for the post-intervention survey.

#### Qualitative data processing and management

Each focus group discussion is audio-recorded using two digital recording devices, and notes are taken by a research assistant. Audio-recordings and notes are transcribed verbatim into Microsoft Word by an independent commercial company. A member of the research team reads through the interview data to address spelling mistakes, inconsistent spacing and formatting, and to check for and resolve any possible transcription errors. Cleaned transcriptions are then imported into NVivo version 11 for the electronic management of analyses. The dataset will be digitally secured with a password, shared only among the members of the analysis team.

One-on-one in-depth interviews are also recorded on a digital recording device. Audio-recordings are transcribed verbatim to Microsoft Word by a research officer at the CHSR&D. A member of the research team will read through the interview data to address spelling mistakes, inconsistent spacing and formatting, and to check for and resolve any possible transcription errors. Any information that identifies a particular hospital, person, or participant will be replaced by unique identifying numbers. Transcribed data are then imported into to NVivo version 11 for the electronic management of analyses. The dataset will be digitally secured with a password, shared only among analysis team members.

## Data analyses

### Quantitative data (SPIRIT point 20a; 20b)

The analysis of quantitative data involves three steps.

1. A number of scales were developed to measure HCWs’ internal and external stigma towards HIV and TB. As a method for theory testing, the scales were validated through confirmatory factor analysis (CFA) using Mplus [43]
  A detailed description of the validation of the scales has been published [21], but to summarise:
  The questionnaire items designed to measure HIV and TB stigma and which held a sufficient factor loading (> 0.5) were included in the scales, as they reflected the theoretical construct [44, 45]. Further, to test how the constructed scales fit the data using model fit indices (the comparative fit index (CFI), the Root Mean Square Error of Approximation (RMSEA), and the Standardised Root Mean Square Residual (SRMR)). A scale was considered reliable if it had a Cronbach’s alpha of above 0.7 [46]. Since the HCWs belong to very different professional categories, measurement invariance was tested among these groups when comparisons in effects or means were done. To compare regression coefficients between the groups, the meaning of the measurement (i.e. the factor loadings) must be invariant across groups; this is referred to as metric invariance. To compare stigma levels, the factor means and intercepts also need to be invariant, this is referred to as scalar invariance [47, 48].
  The baseline data were further analysed with different SEM models, since they could integrate the latent stigma scales. Factors associated with HIV and TB stigma were explored. In terms of a primary (measurement) outcome—scientific information was generated on the extent and sources of HIV- and TB-related stigma among HCWs in the Free State. It is important to note, for reasons explained later, in the section on ‘Monitoring’, these measurement results must be read with caution.
  Analyses of data in terms of the secondary (intervention) outcome—the environment in with HCW’s seek care—is ongoing. So far, two articles have been submitted. In one, a model confirmed the hypothesis that higher stigma levels among HCWs are associated with lower use of occupational health units for HIV and TB services. In another, binominal logistic regression was used to explore factors associated with HCWs’ fear of contracting TB and HIV in the workplace
2. When the post-intervention data have been gathered, the second step of the analysis will be to apply linear mixed modelling to analyse changes in HIV and TB stigma between the intervention and the control group. The within-subject correlation between the two time points can then be accounted for by introducing a random effect into the model [49]. The stigma measurements will have to be metric invariant between the intervention and the control group and scalar invariant between the different time points (pre- and post-assessment) [46, 47]
3. SEM will allow us to test path models that optimally reflect the complex interrelationships between our different key concepts and the HIV and TB stigma between HCWs in the healthcare setting [43]. Therefore, in addition to estimating the intervention effects, the complex pathways to what might cause the effects can be explored through path and mediation analysis. These methods can also assess whether a reduction in stigma over time also could influence the secondary outcomes

### Qualitative data

All qualitative data will be entered into NVivo (qualitative data-management software) and two experienced analysts will conduct a thematic analysis—where text segments are assigned basic codes, organised into categories, and then explored for relationships and overall themes. Transcripts will first be read and re-read to establish a preliminary coding structure. Two researchers will then independently code the scripts using the pre-determined (a priori) codes, but also adding new, emergent codes. Coding will then be interrogated to establish inter-coder reliability, but also with a view to reaching conceptual alignment on existing and emerging codes. The goal of this in-depth analysis is to uncover the mechanisms (*why* and *how*) through which interventions have impacted, or failed to impact, stigma levels in the hospitals.

## Monitoring

In line with the MoU between the CHSR&D (as the implementer of the research) and the FSDoH (as the government department governing the public health facilities in which the research is being conducted), a special steering committee, made up of delegates from both institutions, was convened (SPIRIT point 21a). The committee is independent of the sponsor/funder. The CHSR&D and CELLO keep the committee informed on the progress of the trial via email updates and written progress reports submitted to the committee and to the FSDoH Research Directorate. The CHSR&D also brings any emerging problems or requirements to the attention of the committee, which is tasked to find and agree on appropriate solutions (SPIRIT point 5d).

(SPIRIT points 22, 23) The Memorandum of Agreement (MoU) between the CHSR&D and the FSDoH makes allowance for an outside assessor to appraise the trial mid-way through its implementation and to report any adverse events or unintended negative effects of trial interventions or trial conduct. The assessor is appointed by the FSDoH and is independent from the investigators and the sponsor/funder. In addition, complaints and concerns can also be lodged with the Trial Steering Committee, referred to earlier: it has representatives from different tiers within the FSDoH, including hospital CEOs. Finally, the CHSR&D as key implementer maintains close contact with hospital management and keeps channels of communication open for any dissatisfactions to be aired.

Key to monitoring is to establish whether it is ethical to continue the trial. The three project partners—the FSDoH, CHSR&D, and CELLO—must mutually decide this in consultation with one another and the Trial Committee (SPIRIT point 21a). If very low stigma levels were found at baseline, then intervention would not be warranted. Key results supporting our decision to proceed to the intervention phase are appended (Additional file 6); (SPIRIT point 21b). It is vital to note that these results should be read with extreme caution and are not for dissemination beyond this current article. Both the pilot study’s survey instrument [21] and the adapted baseline survey instrument [50] demonstrated the validity and reliability of the newly developed stigma scales, exhibiting their ability to measure external and internal HIV and TB stigma in the population of HCWs. However, due to the fact that there is no other similar stigma scale (targeted at the HCW population) at our disposal, it is not possible to compare the measured stigma levels to a benchmark value, rendering statements about the severity of the problem difficult. For this reason, the team decided to calculate simple stigma scores from the developed stigma items: for each list of items measuring one type of stigma, we calculated a percentage demonstrating what proportion of the respondents responded *at least once* to a stigmatising statement. The score thus indicates whether the responding HCW had at least one stigmatising response. This approach does not optimally use the data gathered by the Likert items (as the CFA does) and was only intended to provide us with a rough estimate of stigma levels to assess the trial mid-term.

## Ethics, consent and permissions

This study was granted formal ethical approval from two independent committees (SPIRIT point 24); details provided at the end of the article under ‘Ethics approval and consent to participate’.

(SPIRIT point 25) Further protocol amendments are not anticipated, but should any be required they will be sent to ethics committees for review and approval. In line with the MoU between CHSR&D and FSDoH, approval for changes that have ethical implications will also be sought from the FSDoH via the Trial Steering Committee and the FSDoH Research Directorate. Such changes will also be submitted to the trial registry. Authors will submit updates to the journal *Trials* if this is recommended practice.

Both ethics committees follow international standards for research with human subjects, inter alia, the 2002 version of the Declaration of Helsinki. All participants are informed in full about the research, what their participation involves, and what their rights are—for instance that participation is voluntary, that they are free to withdraw from the study, and that there are no costs or benefits to them personally for participating. Those who decide to participate must sign their consent; Model information sheets and consent forms for the survey, the focus group discussions, and the one-on-one interviews are appended (Additional file 3). Fieldworkers who are trained, and ethically sensitised, negotiate each consent in one of three local languages, depending on which the participant prefers (SPIRIT point 26a). It is acknowledged that stigma is a sensitive issue and that the research could evoke emotional discomfort for some participants. Participants experiencing adverse effects from the trial are referred to a specialist division within the FSDoH—the Employee Assistance Programme (EAP)—which employs psychologists and social workers and is mandated to support the welfare of HCWs (SPIRIT point 30).

Although participants have to provide their full names and contact details on the consent form, proper measures are taken to protect their confidentiality. Their names are replaced with a unique number that is pasted onto their consent forms, their completed survey questionnaires, and the participant list kept by the fieldworkers for that day. Consent forms are then separated from the questionnaires. At the end of each day, the lists, consent forms, and completed questionnaires are managed by fieldwork team leaders. The fieldwork teams meet with a CHSR&D PI on a weekly basis to check progress, discuss any problems, and hand in completed questionnaires.

(SPIRIT point 27) All the paperwork then proceeds to the CHSR&D premises for data capturing. The Centre has a separate and secure data room where all physical records and questionnaires are kept locked away. Electronic data—including the list of participant names matched to their unique identifying numbers—are captured on password-protected computers and stored in password-protected files. The electronic data used in analyses are thus entirely anonymised. Only the PI in charge of data collection keeps the list with the participant names and their unique numbers; it is essential to have this information because the same participants are followed up in the post-intervention survey. Trial data are kept in these secure conditions for a period of 10 years, after which they are destroyed.

Access to the final trial dataset is limited to investigators who are project team members and who work in the two research partner institutions: the CHSR&D and CELLO (SPIRIT point 29). Principal investigators declare that they have are no financial or other competing interests for the overall trial or any of the study sites (SPIRIT point 28).

In addition, to ethics approval, formal permission to conduct the (baseline) study and its interventions in Free State public health facilities was obtained from the FSDoH (Additional files 7 and 8).

## Dissemination of results and findings (SPIRIT point 31a)

*Firstly*, we will share our findings with the international scientific community via peer-reviewed publications in journals with an SCI-impact factor (e.g. *Clinical Infectious Diseases*, *Social Science & Medicine*, *International Journal of Tuberculosis and Lung Disease*, etc.). These peer-reviewed publications will be co-authored by project team members, occasionally including co-authors who contributed significantly to the content or analytical bases of an article; professional ‘ghost’ writers will never be used (SPIRIT point 31b). We will also present the results at (inter)national conferences (e.g. *Union World Conference on Lung Health*; *International Conference on AIDS & STIs in Africa*, etc.).

*Secondly*, we will share our findings with the healthcare workforce (e.g. hospital CEOs, HCWs, occupational health representatives) in the eight hospitals where the research was conducted.

*Finally*, we will present our results to the FSDoH in a study report that includes a list of evidence-based recommendations to combat HIV and TB stigma in South African health facilities. In this way we hope to contribute to the development and implementation of effective and sustainable stigma-reduction policies and strategies.

As noted at the end of the article in the section 'Availability of data and materials', access to the dataset is restricted, at this point in time, to the two research institutions (CHSR&D and CELLO) and the project research team members. The same applies to the statistical code (SPIRIT point 31c). In due course, these may be made available by the corresponding author and co-PI (AR), or the CELLO PI (EW) on reasonable request, and once clearance is obtained from the UFS, UA, and FSDoH.

## Discussion

The project encountered some unexpected delays, stemming from a delay at the funding body, VLIR-UOS. The project was granted funding in November 2014. It was communicated that the project (requiring a total of 4 years of research activities) should start after the signing of the Royal Decree (KB) (10 April) and before 12 December 2015. However, we also received an email explaining that all VLIR-UOS projects would be subject to a new regulatory framework which entailed an important consequence for the current project: Phase I – originally scheduled to last 24 months – should now end before 31 December 2016. This would shorten Phase I of the project from 24 to 20 months. In addition, the final contract (which can only be signed after the KB is published) releasing the funding was only finished in June 2015, again cutting on the duration of Phase I: we now only had 18 months to execute 24 months of research activities – we also funded the first months of activities from our own resources as the funds only became available in November 2015.

The project team has done its best to minimise the impact of this shortened time span on the research activities. Nonetheless, we could not implement the intervention in Phase I (as originally planned). This caused some funding issues as the funds for this intervention were budgeted in Phase I and could not be transferred to Phase II. We had to adapt the interventions and consulted the Free State Department of health and the intervention hospitals to jointly redesign the interventions. This consultative process ended in November 2016 with the signing of a Memorandum of Understanding between the implementing partners, and the issue of a new letter of permission from the FSDoH (Additional file 8); (all preliminary research activities, including the baseline survey, were granted permission under an earlier permission letter (Additional file 7)). We were thus able to start with interventions only in early 2017. Implementation has gone extremely smoothly and the interventions have been very well received. We are, therefore, on track to successfully test the intervention in the cluster RCT follow-up survey in 2018.

There were also on-the-ground changes in the research context, which necessitated adaptations to the original protocol (version 1). On 1 September 2016, South Africa moved to a Universal Test and Treat (UTT) strategy where HIV testing and treatment is made widely available, for free, in community settings (including commercial pharmacies). UTT is in line with South Africa’s National Development Plan (NDP) 2030, the UN Sustainable Development Goals, and UNAIDS 90-90-90 targets of 2020 [51]. The implication for our study was that there would no longer be as much emphasis on encouraging HCWs to access HIV and TB testing and treatment on site, in their hospitals, via their occupational health units (OHUs). This shift, and the continuing shortage of human resources for health in the public health system, resulted in two out of the four intervention hospitals no longer having a functional OHU. This shift meant that one of our sub-objectives had to be reformulated. Accordingly, ‘To improve use of occupational health units (OHUs) for HIV and TB screening and treatment in intervention hospitals’, was replaced by ‘To improve the environment in which healthcare workers seek care’.

## Trial status

An application for funding was submitted in October 2014 to VLIR-UOS (Vlaamse Interuniversitaire Raad—Flemish Interuniversity Council). It went through a rigorous external review process; enquiries about the funding and the trial sponsor can be directed to the VLIR-UOS contact person Christophe Goossens, Programme Officer South: Cambodia, Vietnam, South Africa and Mozambique; (Tel: + 32 2550 19 65; email: christophe.goossens@vliruos.be) (SPIRIT point 5b).

Funding was granted for 4 years, and the first tranche was released in Year 1, November 2015. Recruitment (baseline) began in Year 2, February 2016, and recruitment for the first phase of interventions began in Year 3, March 2017. We anticipate that recruitment will be completed by Year 4, August 2018—after the post-intervention survey and subsequent qualitative work. (SPIRIT point 4; see also ‘Section 13.5’).

Changes to the original protocol (version 1 of 16 September 2015) were approved by ethics; this article is based on the final protocol (version 2 of 7 December, 2016) (Additional file 9 shows both versions); (SPIRIT point 3). Permission to proceed with the study (baseline) was granted by the Free State Department of Health in July 2015, permission for the interventions was granted by in February 2017 (respectively, Additional files 7 and 8).

## Additional files

Additional file 1: Standard Protocol Items: Recommendations for Interventional Trials (SPIRIT) Checklist. (DOC 122 kb)

Additional file 2: Stigma randomised controlled trial (RCT) sites. (DOCX 67 kb)

Additional file 3: Model consent forms. (DOCX 885 kb)

Additional file 4: Number of change agents for trainings. (DOCX 18 kb)

Additional file 5: HIV and TB stigma scale items. (DOCX 20 kb)

Additional file 6: Preliminary baseline results. (DOCX 19 kb)

Additional file 7: FSDoH Permission Baseline phase 2015. (DOCX 83 kb)

Additional file 8: FSDoH Permission Intervention phase 2017. (DOCX 47 kb)

Additional file 9: Proposal original 2015 plus Amendments 2016. (DOC 164 kb)

## Abbreviations

AU: Antwerp University
CELLO: Research Centre for Longitudinal & Life Course Studies
CEO: Chief executive officer
CHSR&D: Centre for Health Systems Research & Development
FSDoH: Free State Department of Health
HCW: Healthcare worker
HIV/AIDS: Human immunodeficiency virus (HIV)/acquired immune-deficiency syndrome (AIDS)
IRB: Institutional Review Board
MoU: Memorandum of Agreement
OH: Occupational health
PI: Principal investigator
SBCC: Social and Behavioural Change Communication
TB: Tuberculosis
UA: University of Antwerp
UFS: University of the Free State
VLIR-UOS: Vlaamse Interuniversitaire Raad—Flemish Interuniversity Council
