# Supplementary figures and images for: Towards a health-enabling working environment - developing and testing interventions to decrease HIV and TB stigma among healthcare workers in the Free State, South Africa: study protocol for a randomised controlled trial
Source: Trials. 2018 Jul 4;19:351. doi: 10.1186/s13063-018-2713-5 (PMC6031140; doi:10.1186/s13063-018-2713-5)

Number of hospitals in each Arm = 4.

Randomly selected RCT hospitals are shown in red


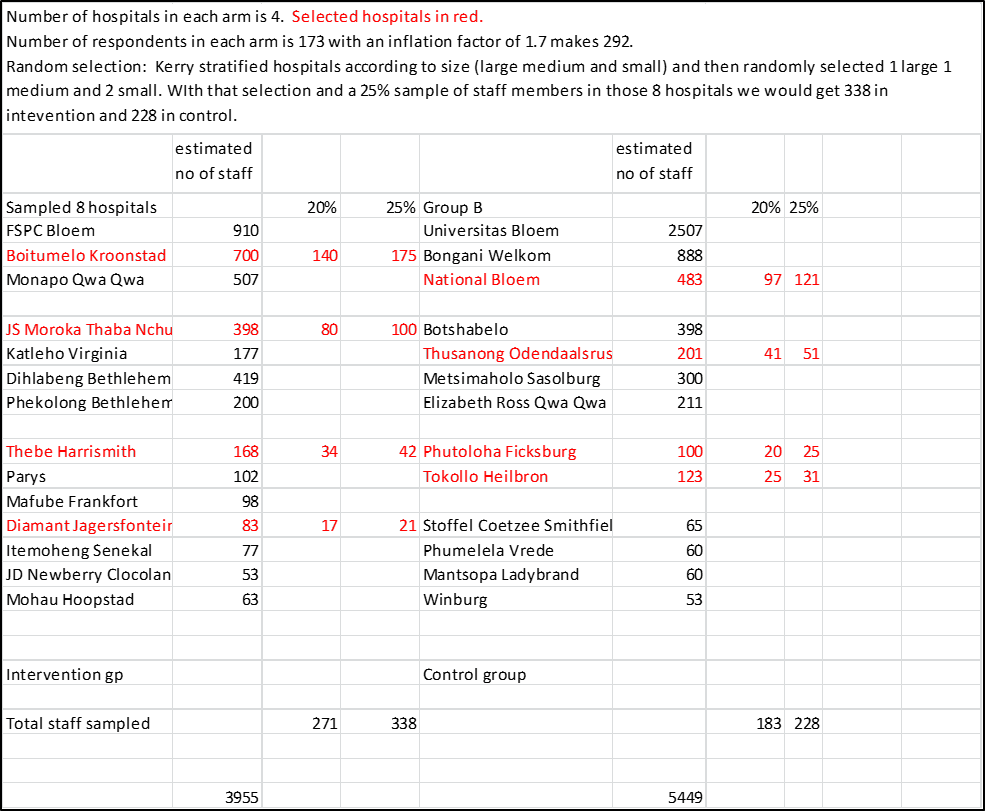

Supplement: Supplementary file 2 — Stigma randomised controlled trial (RCT) sites. (DOCX 67 kb) [file 13063_2018_2713_MOESM2_ESM.docx]
