# Supplementary material for: Towards a health-enabling working environment - developing and testing interventions to decrease HIV and TB stigma among healthcare workers in the Free State, South Africa: study protocol for a randomised controlled trial
Source: Trials. 2018 Jul 4;19:351. doi: 10.1186/s13063-018-2713-5 (PMC6031140; doi:10.1186/s13063-018-2713-5)
Supplement: Supplementary file 3 — Model consent forms. (DOCX 885 kb) [file 13063_2018_2713_MOESM3_ESM.docx]

2015

Dear Sir/Madam [Survey]

**You are kindly invited to participate in a survey. Before you decide whether to take part, here is why we are doing this study and what it involves:**

A team from the Centre for Health Systems Research & Development (CHSR&D), University of the Free State and the University of Antwerp, is carrying out a study to see if implementing a TB and HIV stigma reduction campaign reduces stigma in the workplace. The study is funded by VLIR - OUS.

This study will be conducted in all 8 public hospitals in the Free State. The hospitals will be randomly assigned to control and intervention groups. The study will be conducted over a four-year period, so that we can see if conditions improve over time.

Your name was randomly selected from a list of all hospital employees. Your participation will help a lot in making this assessment. If you agree, you will be asked to complete a questionnaire on issues surrounding TB and HIV stigma and confidentiality in the workplace. We will again contact you in about two and a half years’ time to complete the same questionnaire.

Your participation is entirely voluntary and you have the right to withdraw at any stage in the process without any consequences regarding your work or status of employment. You have the right to ask, and have answered to your satisfaction, any questions about the research study. Your responses will **not** be linked in any way with your name or appear in any reports. We will need you to provide your name and sign this consent form, but the form will be kept safely in a locked cabinet in the office of a UFS researcher and no one from the FSDoH will have access to it. And the consent form will be kept separate from your questionnaire, so once you have filled in the questionnaire **it will be impossible to link your responses to your name**. **As researchers we will do everything in our power to protect your confidentiality**

Note that you will receive no remuneration for participating in the study nor will there be any costs payable by you.

Should you require any additional information concerning this study at any time, please contact the following researcher:

Name: Phone number:

**Dr Asta Rau 051 – 4012181 or 4013750**

**Dr Michelle Engelbrecht 051 – 4012181 or 4013256**

If you have any queries related to ethics, you may contact:

Ethics Committee: Health Sciences

Block D, Deans Division, Room D104

PO Box 2339 (Internal Box G40)

Bloemfontein

Tel: 051 – 401 7795

e-mail: EthicsFHS@ufs.ac.za

**I, _________________________________________________________________________**

**[FULL NAME OF RESPONDENT IN BLOCK LETTERS]**

Have read and understood all the above information;

was given the opportunity to discuss this information and ask questions;

volunteer to take part in this study; and

confirm that I have received a copy of this consent form.

**Signature of respondent: Date:**

**___________________________ ____________________**

**Signature of fieldworker: Date:**

**______________________________ ____________________**


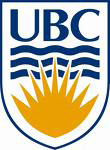


2015

Dear Sir/Madam [Focus group discussion participant]

**You are kindly invited to participate in a focus group discussion. Before you decide whether to take part, here is why we are doing this study and what it involves:**

A team from the Centre for Health Systems Research & Development (CHSR&D), University of the Free State and the University of Antwerp, is carrying out a study to see if implementing a TB and HIV stigma reduction campaign reduces stigma in the workplace. The study is funded by VLIR - OUS

This study will be conducted in all 8 public hospitals in the Free State. The hospitals will be randomly assigned to control and intervention groups. The study will be conducted over a four-year period, so that we can see if conditions improve over time.

Your participation will help a lot in making this assessment. If you agree, you will be asked to participate in a focus group discussion on TB and HIV stigma and confidentiality in the workplace. We plan to audio-record the session and transcribe the recording into text.

Your participation is entirely voluntary and you have the right to withdraw at any stage in the process without any consequences regarding your work or status of employment. You have the right to ask, and have answered to your satisfaction, any questions about the research study and the focus group. We will not use your name during the recording and your name will not appear on any of the transcripts or in the reports. The audio-recording will be transcribed by one person, working at UFS, who is appropriately trained. The digital recording and transcript will be kept in a password-protected file. We will need you to provide your name and sign this consent form, but the form will be kept safely in a locked cabinet in the office of a UFS researcher and no one from the FSDoH will have access to it.

As researchers we will do everything in our power to protect your confidentiality. You should be aware, however, that we have no control over what group members say *outside* of the group. What we can do as researchers to protect you in this case is to set group norms before we begin the focus group — one of the norms is to not discuss, outside of the group, anything that is said during the focus group. By agreeing to participate you would be undertaking to uphold this courtesy to all your group members.

Note that you will receive no remuneration for participating in the study nor will you need to pay any costs.

Should you require any additional information concerning this study, you may contact the following researcher:

Name: Phone number:

**Dr Asta Rau 051 – 4012181 or 4013750**

**Dr Michelle Engelbrecht 051 – 4012181 or 4013256**

If you have any queries related to ethics, you may contact:

Ethics Committee: Health Sciences

Block D, Deans Division, Room D104

PO Box 2339 (Internal Box G40)

Bloemfontein

Tel: 051 – 401 7795

e-mail: EthicsFHS@ufs.ac.za

**I, _________________________________________________________________________**

**[FULL NAME OF RESPONDENT IN BLOCK LETTERS]**

Have read and understood all the above information;

was given the opportunity to discuss this information and ask questions;

volunteer to take part in this study; and

confirm that I have received a copy of this consent form.

**Signature of respondent: Date:**

**___________________________ ____________________**

**Signature of researcher: Date:**

**______________________________ ____________________**

2015

Dear Sir/Madam [One-on-one interview with Health care workers with TB and/or HIV/AIDS]

**You are kindly invited to participate in an interview. Before you decide whether to take part, here is why we are doing this study and what it involves:**

A team from the Centre for Health Systems Research & Development (CHSR&D), University of the Free State and the University of Antwerp, is carrying out a study to see if implementing a TB and HIV stigma reduction campaign reduces stigma in the workplace. The study is funded by VLIR - OUS

This study will be conducted in all 8 public hospitals in the Free State. The hospitals will be randomly assigned to control and intervention groups. The study will be conducted over a four-year period, so that we can see if conditions improve over time.

Your participation will help a lot in making this assessment. If you agree, you will be asked to participate in an interview on the use of the unit for TB and/or HIV testing and treatment as well as on issues surrounding TB and HIV stigma and confidentiality in the workplace. We plan to audio-record the interview and transcribe the recording into text.

Your participation is entirely voluntary and you have the right to withdraw at any stage in the process without any consequences regarding your work or status of employment. You have the right to ask, and have answered to your satisfaction, any questions about the research study and the interview. We will not use your name during the recording and your name will not appear on any of the transcripts or in the reports. The audio-recording will be transcribed by one person, working at UFS, who is appropriately trained. The digital recording and transcript will be kept in a password-protected file. We will need you to provide your name and sign this consent form, but the form will be kept safely in a locked cabinet in the office of a UFS researcher and no one from the FSDoH will have access to it. **As researchers we will do everything in our power to protect your confidentiality.**

Note that you will receive no remuneration for participating in the study nor will you need to pay any costs.

Should you require any additional information concerning this study, you may contact the following researcher:

Name: Phone number:

**Dr Asta Rau 051 – 4012181 or 4013750**

**Dr Michelle Engelbrecht 051 – 4012181 or 4013256**

If you have any queries related to ethics, you may contact:

Ethics Committee: Health Sciences

Block D, Deans Division, Room D104

PO Box 2339 (Internal Box G40)

Bloemfontein

Tel: 051 – 401 7795

e-mail: EthicsFHS@ufs.ac.za

**I, _________________________________________________________________________**

**[FULL NAME OF RESPONDENT IN BLOCK LETTERS]**

Have read and understood all the above information;

was given the opportunity to discuss this information and ask questions;

volunteer to take part in this study; and

confirm that I have received a copy of this consent form.

**Signature of respondent: Date:**

**___________________________ ____________________**

**Signature of researcher: Date:**

**______________________________ ____________________**

2017

Dear Sir/Madam [Workshop]

**You are kindly invited to participate in a workshop. Before you decide whether to take part, here is why we are doing this study and what it involves:**

A team from the Centre for Health Systems Research & Development (CHSR&D), University of the Free State and the University of Antwerp, is carrying out a study to see if implementing a TB- and HIV-stigma reduction campaign reduces stigma in the workplace. The study is funded by VLIR-OUS.

Our research is being conducted in 8 Free State hospitals. The 8 hospitals were randomly selected from all the hospitals in the Free State. Out of the 8 hospitals we again randomly selected 4 that will get stigma reduction interventions (called the intervention group) and 4 that will *not* get the interventions (called the control group). The hospitals in the intervention group are: Boitumelo; JS Moroka; Thebe; and Diamant.

There are several layers to the interventions. The main socio-behavioural intervention is the stigma-reduction workshops. Our stigma-reduction workshops are targeted at *change agents*. The stigma reduction changes that need to happen are mainly changes in knowledge and attitudes (and hopefully, behaviours)—and this needs change agents to *personally influence* others in the workplace by being *role models* for stigma reduction.

You were identified as a change agent in a participatory way, by asking people in prominent positions and jobs question such as: “Who are the people in this hospital that other people look up to? Who do they go to for help? Who is an HIV-champion in the hospital? Who is likely to want, and be able, to help reduce HIV- and TB-stigma in this hospital?”

We are approaching you, as a person who has been identified as a potential change agent, to ask if you are interested in training on HIV- and TB-stigma, and interested in helping to reduce it in your hospital. If you accept the invitation and attend the training you will be asked to nominate other change agents, who we will then approach for the second round of training sessions in 2017.

Participants will be trained in the language they prefer and will come from 3 main groups: clinical staff (e.g. nurses’ allied health workers; pharmacists; etc.); administration and management (e.g. people from Human Resources and Finances; secretaries; etc.); and support staff (e.g. people in housekeeping; messengers; cleaners; etc.).

At the end of the training you should be able to: explain what stigma is; differentiate between internal and external stigma; identify the causes, forms and effects of stigma; explain what it feels like to stigmatise and be stigmatised; explain healthcare workers’ rights and responsibilities that are key to HIV- and TB-stigma and its reduction in the workplace; implement S.W.A.T (a series of steps to help change agents to stop HIV and TB stigma in the workplace); and identify some easy strategies that you can use to combat stigma in the workplace and plan how you will implement your ideas.

Training will be available in the three main languages of the province (English, Afrikaans & Sesotho) and you can choose which language you feel most comfortable with. The workshop will take approximately 4 hours and there are no risks involved. Some participants might feel a bit of discomfort during the workshop due to the sensitive nature of stigma and being stigmatised. We will ask you to complete an evaluation form at the end of the workshop that will assist us to improve our workshop intervention.

We will follow-up with you once the first set of trainings are completed at all four hospitals. It might be in a quick chat with you as individual or in a focus group discussion with the group members who trained with you. The aim of following-up is to see *if* and *how* the change agents implemented their plans to address TB- and HIV-stigma, and what opportunities and difficulties they experience.

Your participation is entirely voluntary and you have the right to withdraw at any stage in the process without any consequences regarding your work or status of employment. You have the right to ask, and have answered to your satisfaction, any questions about the research study. Your responses will **not** be linked in any way with your name or appear in any reports. We will need you to provide your name and sign this consent form, but the form will be kept safely in a locked cabinet in the office of a UFS researcher and no one from the FSDoH will have access to it. The consent form will be kept separate from your evaluation form, so once you have filled in the evaluation form **it will be impossible to link your responses to your name**. **As researchers we will do everything in our power to protect your confidentiality**

Note that you will receive no remuneration for participating in the study nor will there be any costs payable by you.

Should you require any additional information concerning this study at any time, please contact the following researcher:

Name: Phone number:

**Dr Asta Rau 051 – 4012181 or 4013750**

**Dr Michelle Engelbrecht 051 – 4012181 or 4013256**

If you have any queries related to ethics, you may contact:

Ethics Committee: Health Sciences

Block D, Deans Division, Room D104

PO Box 2339 (Internal Box G40)

Bloemfontein

Tel: 051 – 401 7795

e-mail: EthicsFHS@ufs.ac.za

**I, _________________________________________________________________________**

**[FULL NAME OF RESPONDENT IN BLOCK LETTERS]**

Have read and understood all the above information;

was given the opportunity to discuss this information and ask questions;

volunteer to take part in this study; and

confirm that I have received a copy of this consent form.

**Signature of respondent: Date:**

**___________________________ ____________________**

**Signature of witness: Date:**

**______________________________ ____________________**
