# Supplementary material for: Towards a health-enabling working environment - developing and testing interventions to decrease HIV and TB stigma among healthcare workers in the Free State, South Africa: study protocol for a randomised controlled trial
Source: Trials. 2018 Jul 4;19:351. doi: 10.1186/s13063-018-2713-5 (PMC6031140; doi:10.1186/s13063-018-2713-5)
Supplement: Supplementary file 4 — Number of change agents for trainings. (DOCX 18 kb) [file 13063_2018_2713_MOESM4_ESM.docx]

**Diamant Hospital (this is the calculation for 1 round of training; x 2 for the full dose)**

| **Category** | **Number** | **30% Sample** | **35% Sample** | **Training numbers** | **Training sessions** | **People per training session** |
| --- | --- | --- | --- | --- | --- | --- |
| Doctors | 1 | 0 | 0 | 17 | 1 | 20 |
| Nurses | 43 | 13 | 15 |  |  |  |
| Allied | 5 | 2 | 2 |  |  |  |
| Management & Admin | 9 | 3 | 3 | 3 |  |  |
| Support | 28 | 8 | 10 | 10 | 1 | 10 |
| **Total** | **86** | **26** | **30** | **30** | **2** |  |

**Thebe Hospital (this is the calculation for 1 round of training; x 2 for the full dose)**

| **Category** | **Number** | **30% Sample** | **35% Sample** | **Training numbers** | **Training sessions** | **People per training session** |
| --- | --- | --- | --- | --- | --- | --- |
| Doctors | 1 | 0 | 0 | 32 | 2 | 20 |
| Nurses | 76 | 23 | 27 |  |  |  |
| Allied | 15 | 4 | 5 |  |  |  |
| Management & Admin | 22 | 7 | 8 | 8 |  |  |
| Support | 33 | 10 | 11 | 11 | 1 | 11 |
| **Total** | **147** | **47** | **51** | **51** | **3** |  |

**Boitumelo Hospital (this is the calculation for 1 round of training; x 2 for the full dose)**

| **Category** | **Number** | **30% Sample** | **35% Sample** | **Training numbers** | **Training sessions** | **People per training session** |
| --- | --- | --- | --- | --- | --- | --- |
| Doctors | 32 | 10 | 11 | 109 | 6 | 18-19 |
| Nurses | 252 | 75 | 88 |  |  |  |
| Allied | 29 | 9 | 10 |  |  |  |
| Management & Admin | 62 | 19 | 22 | 22 | 1 | 22 |
| Support | 206 | 62 | 72 | 72 | 4 | 18 |
| **Total** | **581** | **175** | **203** | **203** | **11** |  |

**JS Moroka Hospital (this is the calculation for 1 round of training; x 2 for the full dose)**

| **Category** | **Number** | **30% Sample** | **35% Sample** | **Training numbers** | **Training sessions** | **People per training session** |
| --- | --- | --- | --- | --- | --- | --- |
| Doctors | 11 | 3 | 4 | 56 | 3 | 18-19 |
| Nurses | 138 | 41 | 48 |  |  |  |
| Allied | 12 | 4 | 4 |  |  |  |
| Management & Admin | 47 | 14 | 16 | 16 | 1 | 16 |
| Support | 130 | 39 | 46 | 46 | 3 | 15-16 |
| **Total** | **338** | **101** | **118** | **118** | **7** |  |
