# Supplementary material for: Towards a health-enabling working environment - developing and testing interventions to decrease HIV and TB stigma among healthcare workers in the Free State, South Africa: study protocol for a randomised controlled trial
Source: Trials. 2018 Jul 4;19:351. doi: 10.1186/s13063-018-2713-5 (PMC6031140; doi:10.1186/s13063-018-2713-5)
Supplement: Supplementary file 5 — HIV and TB stigma scale items. (DOCX 20 kb) [file 13063_2018_2713_MOESM5_ESM.docx]

**HIV & TB Stigma Scales in the baseline survey**

**All items on all scales have 4 response options: Strongly Agree; Agree; Disagree; Strongly Disagree**

**The general climate of *HIV* stigma in this hospital**

1. Some other healthcare workers in this hospital tend to neglect patients who they think may have HIV
2. Some other healthcare workers in this hospital talk badly about patients who they think may have HIV
3. Some other healthcare workers in this hospital look down on patients who they think may have HIV
4. Some other healthcare workers in this hospital feel uncomfortable being around patients who they think may have HIV
5. Some other healthcare workers in this hospital are afraid of catching HIV from patients

**The general climate of *TB* stigma in this hospital**

1. Some other healthcare workers in this hospital tend to neglect patients who they think may have TB
2. Some other healthcare workers in this hospital talk badly about patients who they think have TB
3. Some other healthcare workers in this hospital look down on patients who they think may have TB
4. Some other healthcare workers in this hospital feel uncomfortable being around patients who they think may have TB
5. Some other healthcare workers in this hospital are afraid of catching TB from patients

***HIV* stigma among *healthcare workers***

1. Some of my co-workers in this hospital look down on healthcare workers who they think may be HIV-infected
2. I would feel comfortable being close friends with a healthcare worker who is known to be HIV-positive
3. If I was HIV-positive, I would worry that some co-workers might avoid touching me
4. There are healthcare workers who make negative remarks about the health of co-workers who are involved in HIV care and treatment
5. I would feel comfortable having healthcare workers who are known to be HIV-positive working closely with me in my job
6. Healthcare workers who have HIV should not feel guilty about it
7. HIV-positive healthcare workers can be good role models in the workplace
8. Doctors and nurses with HIV who are otherwise in good health should continue to practise medicine
9. Some healthcare workers who are suspected of having HIV get rejected by others in the workplace
10. Other healthcare workers in this hospital are afraid of catching HIV from colleagues who care for HIV-positive patients
11. If I had HIV I would feel comfortable disclosing to some of my co-workers
12. If I was diagnosed with HIV, I would be afraid that some co-workers might blame me for being infected
13. As a healthcare worker I would feel it was my fault if I was infected with HIV
14. If I had HIV I would avoid making new friends at my workplace
15. Some healthcare workers in this hospital are known to give extra support to colleagues with HIV
16. Some healthcare workers in this hospital educate co-workers who stigmatise people living with HIV
17. Some healthcare workers in this hospital are doing something to stop stigma in the workplace

***TB* stigma among healthcare workers**

1. Hhealthcare workers who are suspected of having TB are stigmatised in this hospital
2. Some healthcare workers in this hospital avoid contact with co-workers who they think may have TB
3. If I was diagnosed with TB I would not need to feel shame
4. As a healthcare worker I would feel it was my fault if I was infected with TB
5. Some healthcare workers in this hospital would not want to eat or drink with a co-worker who they think has TB
6. If I was diagnosed with TB I would feel comfortable to tell some of my co-workers
7. Some healthcare workers in this hospital are stigmatised when others find out that they have gone for TB screening
8. I do not want to work together with co-workers who are on TB treatment
9. I have noticed that some other healthcare workers in this hospital feel uncomfortable to work near co-workers with TB
10. If I was diagnosed with TB I would feel alone in my workplace
11. I am cautious of co-workers who are on TB treatment
12. I If I think a co-worker has TB I will avoid eating or drinking in the same room

**HIV and TB Stigma *combined***

1. TB is a sign that someone has HIV
2. Someone with TB has probably also got HIV
3. TB symptoms make HIV more noticeable
4. Someone who has TB should feel equally guilty about it as someone who has HIV
5. People with TB tend to be treated badly because they may have HIV
6. People are afraid of working together with someone who has TB because they think that the person also has HIV
7. If I was diagnosed with HIV then, in order to hide my HIV status, I would say that I have TB
8. If I was diagnosed with TB I would emphasise that I do not have HIV
9. If I was diagnosed with both TB and HIV I would only tell that I have TB
