# Supplementary material for: Towards a health-enabling working environment - developing and testing interventions to decrease HIV and TB stigma among healthcare workers in the Free State, South Africa: study protocol for a randomised controlled trial
Source: Trials. 2018 Jul 4;19:351. doi: 10.1186/s13063-018-2713-5 (PMC6031140; doi:10.1186/s13063-018-2713-5)
Supplement: Supplementary file 6 — Preliminary baseline results. (DOCX 19 kb) [file 13063_2018_2713_MOESM6_ESM.docx]

**Baseline results informing the decision to continue the trial**

As noted in the main text, due to the fact that there is no other similar stigma‐scale (targeted at the HCW population) at our disposal, it is not possible to compare the measured stigma‐levels to a benchmark value, rendering statements about the severity of the problem difficult.

For this reason, the team decided to calculate simple stigma scores from the developed stigma items: for each list of items measuring one type of stigma, we calculated a percentage demonstrating what proportion of the respondents responded at least once to a stigmatizing statement. The score thus indicates whether the responding HCW had at least one stigmatizing response. This approach does not optimally use the data gathered by the Likert items (as the Confirmatory Factor Analysis does) and was only intended to provide us with a **rough estimate** of the stigma levels to assess the trial mid‐term.

The estimates are as follows:

Respondent's External Stigma towards HIV: 35.1%

Colleagues' External Stigma towards HIV: 45.7%

Respondent's Internal Stigma towards HIV: 69.4%

Respondent's External Stigma towards TB: 43.3%

Colleagues' External Stigma towards TB: 50.3%

Respondent's Internal Stigma towards TB: 46.4%

52.7% of respondents saw at least one action taken by colleagues to *fight stigma*.
