# Supplementary material for: Towards a health-enabling working environment - developing and testing interventions to decrease HIV and TB stigma among healthcare workers in the Free State, South Africa: study protocol for a randomised controlled trial
Source: Trials. 2018 Jul 4;19:351. doi: 10.1186/s13063-018-2713-5 (PMC6031140; doi:10.1186/s13063-018-2713-5)
Supplement: Supplementary file 9 — Proposal original 2015 plus Amendments 2016. (DOC 164 kb) [file 13063_2018_2713_MOESM9_ESM.doc]

**Towards a health-enabling working environment: developing and testing interventions to decrease HIV- and TB-stigma among healthcare workers in the Free State, South Africa**

**This is the proposal (version 2) approved by ethics on 7 Dec 2016.**

**All additions to the original proposal (that was put through ethics in September 2015), are highlighted green; all changes are shown in tracked changes**

**Research team**

| **Local project team:** | Researchers and staff at CHSR&D, University of the Free State, South Africa: Dr Asta Rau (Director), Dr. Michelle Engelbrecht (Deputy Director), Dr Gladys Kigozi (senior researcher), Prof Christo Heunis (senior researcher); André Janse van Rensburg (researcher), Bridget Smit (data manager) Belinda Jacobs (financial administrator). Alfi Moolman (project coordinator/manager) |
| --- | --- |
|  |  |
| **Flemish team:** | CELLO: Prof dr. Edwin Wouters (Promotor); Prof. dr. Dimitri Mortelmans (Director), Caroline Masquillier (researcher) |
|  |  |
| **Free State Department of Health team:** | Dr. Kerry Uebel (Strategic liaison with FSDoH; Researcher-Clinical practitioner responsible for supporting and mentoring Occupational Health nurses in Nurse-Initiated Management of ART and TB treatment). Lucky Nophale (Provincial Occupational Health Unit) |

# Background and rationale for the project

## Background

HIV/AIDS and tuberculosis (TB) have merged into a deadly **co-epidemic** in South Africa. In absolute numbers, the country has the highest number of people living with HIV (6.1 million in 2012) . In addition it has one of the most severe TB epidemics in the world, with the highest prevalence of TB (857 per 100,000 in 2012) and 15,419 reported cases of multi-drug resistant (MDR) TB in 2012 . Both epidemics are intricately intertwined: approximately 73% of TB cases are co-infected with HIV and TB mortality among HIV-positive people was estimated to be 168 per 100,000 inhabitants .

The dual burden of TB and HIV has a severe impact on the South African **healthcare workforce**. Occupational exposure to TB constitutes a major health risk for healthcare workers (HCWs), especially in resource-constrained settings where large patient numbers and resulting over-crowded health facilities combined with poorly implemented infection control strategies render HCWs three times more likely to acquire TB than the general population . Consequently, TB is officially classified as an occupational disease. The HIV epidemic equally affects the workforce because of the mutually reinforcing epidemiology of HIV and TB: estimates of the HIV prevalence among South African HCWs range from 11.5% to 20.0% .

In this context, **workplace health services for TB and HIV/AIDS** are an essential part of any health systems strengthening strategy . Research has demonstrated that providing HIV and TB services to HCWs at work is cost-effective and is preferred by the majority of HCWs . Accordingly, a joint World Health Organization-International Labour Organization-UNAIDS policy document on the provision of TB and HIV prevention and care for HCWs explicitly recommends the on-site availability of such occupational health services for the entire workforce so that full access to HIV and TB prevention, treatment, care and support for this vulnerable group can be attained .

However, a recent review paper demonstrated that HIV- and TB-related **stigma and discrimination** are “key barriers to both the delivery of quality health services by health providers and to their utilization by community members and health providers themselves” . Stigmatization in the healthcare setting can thus have severe implications for HCWs and health facilities when HIV-positive HCWs delay or avoid care, causing increased morbidity and mortality and further strain on an overburdened health system .These factors indicate that the development and testing of stigma-reduction interventions for this important subpopulation is a research priority.

## Proposed action

The proposed project aims to address this research gap by **(1)** scientifically assessing the extent and sources of HIV and TB-related stigma among the healthcare workforce and **(2)** developing and testing evidence-based stigma-reduction interventions in randomly selected public hospitals in the Free State Province of South Africa.

## Steps undertaken

The success/failure of previous stigma-reduction interventions has been difficult to evaluate because of a dearth of appropriate scientific tools to measure stigma in this specific professional setting. Uys et al. (2009) indicated that the majority of papers reporting the intervention outcomes did not include a validated instrument to measure change in stigma over time . The limited number studies that did measure stigma in the healthcare setting focused almost exclusively on (a) stigmatizing attitudes of HCWs towards *patients*; (b) HIV (and *not TB*); and (c) healthcare professionals e.g. doctors, nurses, but not the *entire healthcare workforce* (cleaners, clerks, security, etc.) , creating a clear **need for reliable and validated scales** to measure both HIV and TB stigma among the entire healthcare workforce. Several significant preparatory steps have been undertaken:

- Based on a solid theoretical framework, the current proposal team (CHSR&D-CELLO) worked closely together in 2012-2013 to develop and refine a range of scales to measure the most important dimensions of TB and HIV stigma: **external and internal stigma** . External stigma is directed by HCWs *outwards* towards other HCWs, and internal stigma is directed by HCWs *inwards* towards themselves. The resulting parallel scales – measuring external and internal stigma towards HIV *and* TB – were piloted in Pelonomi Regional Hospital (Free State, South Africa) (**Stigma Score Pilot Study**; ethical clearance: ECUFS NR 192/2012). This study (gathering data on 220 healthcare staff, including staff involved in direct patient care, support staff and administrative staff) was largely funded by the CHSR&D’s own limited resources as an **investment for future research**. Funding has now been secured from VLIR-UOS to execute a TEAM project that builds on and extends the pilot study. Simultaneously, a literature review on stigma-reduction interventions, particularly in healthcare settings, formed the basis for **workshops with key stakeholders**, out of which a series of **stigma-reduction interventions** designed to work at different socio-ecological levels (individual, community, and structural) were proposed and discussed. To support interventions, locally relevant and culturally sensitive **imagery and slogans** have been piloted and refined: these target HIV and TB stigma, as well as promote confidentiality in occupational health units.

# Research methods

In order to address the above-cited research needs, a **randomized control trial** will be conducted to **(1)** scientifically assess the extent and sources of HIV and TB stigmatization among the healthcare workforce and **(2)** refine and test evidence-based stigma-reduction interventions in randomly selected public hospitals in the Free State Province of South Africa.

The groundwork for the proposed study (the sample size estimation (584), power calculations and hospital selection (8 hospitals stratified by size and district and randomly allocated to intervention or control arms) was performed based on a Stigma Score Pilot Study. **Pre-intervention**, a self-administered questionnaire with the Pilot Study’s validated stigma scales will be used to measure stigma and other key variables among randomly selected HCWs in all 8 sites. The **intervention** will comprise of stigma reduction activities at 3 social-ecology levels (individual, community, and structural) in the 4 intervention sites. To evaluate implementation, qualitative data will be collected from purposively selected HCWs midway through and possibly at other stages during the trial. **Post-intervention**, all respondents from baseline will be followed up using a questionnaire with the same scales as the pre-intervention questionnaire but with added questions on interventions. Quantitative and qualitative data will be collected to not only assess the size of the impact but also uncover the processes through which the interventions impact on stigmatization. **The study results** will be presented in workshops, conferences, reports and journal articles. Specific attention will be given to a continuous dialogue with the relevant stakeholders (FSDoH, hospital managers, etc.) to ensure **the optimal dissemination** of our evidence-based recommendations to combat HIV/AIDS and TB stigma to these agents of change.

## Preparatory research activities

### Ethical clearance, DoH permissions, and legal agreements

Formal ethical clearance will be sought from the Ethics Committee in the Faculty of Health Sciences at the UFS and the UA Social Sciences Ethical Clearance Committee.

The proposal will be submitted to the FSDoH Research Protocol Committee for formal approval to conduct the research. Hospital managers will be consulted and informed about the research process and project start date.

A memorandum of understanding/contracts between CHSR&D, CELLO and the FSDoH will be signed.

### Local research capacity

In line with the funder’s (VLIR-UOS) objectives, the cooperation also aims to strengthen research capacity in the South. To reach this result, a short course presented in **a series of workshops** − supplemented with follow-up mentorship − will be organized to share the methodological experience of CELLO (Profs. Wouters and Mortelmans) with CHSR&D staff. Workshops will tackle advanced statistical analysis techniques such as Structural Equation Modelling (SEM), sampling methods, etc.

### Study sample drawn from the healthcare workforce register

The parameters used for sample size calculation were estimated from the Stigma Score Pilot Study. The mean stigma score considered for the control group was 0.85 with a standard deviation of 0.71. The stigma score is expected to be reduced by approximately 24% to 0.65 in the intervention group with a standard deviation of 0.39. Two-tailed test is considered with Type I error of 0.05 and Type II error of 0.10 give 90% power. The coefficient of variation considered for each group is 0.25 and the intra-cluster correlation coefficient is 0.05. The estimated sample size per arm is 173 and the estimated inflation factor (design effect) is 1.7. Therefore the required sample size for this study per arm is approximately 173X1.7≈292 participants. The total number of participants required for this study is 584 (347 respondents in intervention sites and 237 in control sites). A sampling frame for individual respondents (and replacements) in each site will need to be drawn up. Based on previous experience of fieldwork in Free State hospitals, the baseline survey will need 50% oversampling of respondents to allow for loss-to-follow up between baseline and post-intervention surveys.

The participants are clustered within intervention and control facilities. In order to ensure an equal distribution of large and small hospitals across the different districts in both intervention and control arms, hospitals of similar size (based on numbers of staff), and where possible in the same district, are deliberately paired. Within these pairs, a coin toss is used to randomly allocate hospitals to Arm A or Arm B, and then again to allocate the 2 arms to intervention or control. The number of hospitals required is estimated considering the fact that the number of staff members across the hospitals in each group differ. The average number of staff members per hospital considered is 220. The same parameters stated above are used, and the number of hospitals required is estimated to be ≈ ±4 hospitals per group (intervention – control).

## Baseline of the RCT

### A pre-intervention survey is developed

The experimental design requires a baseline assessment of the HIV and TB stigma levels in both the intervention and control hospitals. A pre-intervention survey will be largely based on the HIV/AIDS and TB stigma scales that were developed and recently validated in the Stigma Score Pilot Study (see Section 1.3). There are, however, several sections in the piloted questionnaire (e.g. demographics; HIV and TB knowledge and disclosure) that require adaptation. The stigma scales will remain unchanged. In addition, the survey will also include questions on a range of other key outcome measures, which are related to stigma at the individual, community, and structural levels (e.g. disclosure, confidentiality, willingness to use occupational health units for HIV and TB services, knowledge of HIV and TB; knowledge of Code of Conduct and workers’ rights, infection control). Occupational health nurses will be contacted twice a year to determine the number of health care workers accessing the occupational health clinic for TB screening and treatment and HIV treatment.

### Pre-intervention data is gathered

The stigma survey will be self-administered by specific, randomly selected individuals (and replacements) willing to take part. Respondents with low levels of reading literacy will be gathered into small groups and the fieldworkers will work through the questions verbally and answer any queries from the group; however, respondents will need to fill in the questionnaires themselves. Fieldworkers experienced in health research will be used, and will be given additional training to sensitize them to standardization of research processes. Subsequently, the data will be captured and cleaned.

### Knowledge on the pre-intervention stigma levels is produced

The resulting dataset will be analyzed by means of the appropriate statistical techniques: especially Structural equation modeling (SEM) – using the software package MPlus – to explore the links between the contextual data gathered (e.g. disclosure, confidentiality) and the stigma-scales. SEM allows for incorporating both latent constructs (confirmatory factor analysis) and path analysis in one model: **(1)** the measurement model specifies the relationships between the observed indicators (the items of the stigma scale) and the latent variables (the over-arching concept of stigma); **(2)** the structural model specifies the relationships among the different relevant concepts (e.g. confidentiality and stigma). This combination allows us to create conceptual models which approach the complexity of social reality.

## The stigma-reduction interventions of the RCT are executed

### The stigma-reduction interventions are finalized

As indicated in 1.1 Background of the project (1.3 Steps undertaken), an extensive literature review on possible interventions has been performed. This review revealed that interventions at 3 socio-ecological levels are required: **(1)** individual, **(2)** community and **(3)** structural-social levels. In a series of **workshops with key stakeholders**, we developed a series of stigma-reduction interventions designed to work at these 3 different levels. Locally relevant and culturally sensitive imagery and slogans have also been piloted and refined. The stigma-reduction interventions are thus basically far developed (see below for their content). Before we can implement these interventions, however, we want to finalize the intervention strategy by drafting **a manual** outlining the different interventions at all 3 levels − enabling as precise a replication of the intervention strategy as possible in order to confirm our study findings in different contexts.

### The stigma-reduction interventions are implemented

Adhering to the above-cited manual, we will—in partnership with DoH staff (the ‘champions’ described below)—implement the interventions in all the experimental hospitals. We will take all precautions possible to prevent contamination of the control hospitals. The stigma-reduction interventions target 3 levels. *First* of all, the **individual-level** interventions target **the training of trainers**: in keeping with a Diffusion of Innovation approach , much emphasis is put on recruiting influential and respected ground-level opinion leaders and “champions” to actively promote a stigma-free workforce and workplace. Examples of such potential trainers are Department of Health staff in the Employee Assistance Program (EAP), which is a unit employed specifically to promote PHDP (Positive Health, Dignity and Prevention); occupational health nurses; health & safety representatives; union representatives, and people living with HIV/AIDS. In practice, dynamic and lively training-of-trainers workshops will focus on **(1)** maintaining and promoting confidentiality at occupational health units, **(2)** understanding stigma, and **(3)** strategies to reduce stigma. *Secondly*, the **community-level** interventions will employ **trainers as change agents**: these people are equipped to vertically and horizontally communicate key HIV-TB knowledge and stigma-reduction messages to all hospital staff. In addition, a mass media campaign (e.g. awareness days, posters) within each intervention hospital will be led and run by the facility staff (trainers) with funding from the project budget. At the end, all staff within a facility should be reached by one or more of the stigma-reduction interventions (this can be investigated in the post-intervention survey. see below). *Finally*, the **structural-social level** interventions target **all healthcare staff as well as the hospital managers**. These interventions include **(1)** providing HIV- and TB-related clinical support to OHUs, and where OHUs do not exist to staff doctors and nurses who test and treat facility staff for HIV & TB (Dr Kerry Uebel of the FSDoH will be leading this);**(2)** distribution of information on rights & responsibilities in the healthcare workplace as relevant to HIV- and TB-stigma and **(3)** (optional) regular and consistent messaging from the top management and champions/opinion leaders, communicating ‘Zero Tolerance for non-confidentiality’ and ‘Zero stigma and discrimination’.

## Impact of the stigma-reduction interventions is evaluated in the RCT

### The post-intervention survey is developed

To ensure comparability, the post-intervention survey will resemble the pre-intervention survey, thus including the HIV/AIDS and TB stigma scales as well as a range of other key outcome measures, related to stigma at the individual, community, and structural levels (e.g. disclosure, confidentiality, willingness to use occupational health units for HIV and TB services, HIV/TB knowledge; knowledge of Code of Conduct and workers’ rights). The post-intervention survey will also include a section that measures the reach and appeal of the stigma interventions.

### Post-intervention data is gathered

The same data gathering procedure occurred in the baseline (self-administered survey, verbal support of fieldworkers if needed) will apply. In addition, routine anonymous data on staff utilization of TB-HIV services will be collected from the occupational health units to allow us to objectively assess the impact of the interventions.

However, these **quantitative data** will only allow us to assess the extent of the impact of the interventions, not *how* and *why* they have an impact. **Qualitative data** collection is needed to complement the information of the quantitative data. A mid-term and post-intervention evaluation will involve the following: **(1)** One-on-one interviews will be held with *purposively selected* key implementers and stakeholders in all sites to evaluate the effectiveness of each of the different stigma interventions and their implementation, as well as to distil lessons and recommendations. **(2)** Evaluation will include focus-group discussions with *conveniently sampled* staff at different occupational levels in each of the intervention sites. **(3)** Occupational Health nurses/ staff nurses or doctors providing HIV and TB services to other healthcare workers in their facilities will be asked at the end of the trial to confidentially contact individual staff members who have been treated for TB or HIV to ask if they would be willing to take part in a one-on-one interview with a researcher. All interviews and focus group discussions will be conducted using a semi-structured interview guide, be audio-recorded, translated when necessary, and transcribed.

### Knowledge on the impact of the intervention is produced

Classical **experimental analysis techniques** will be applied to assess the impact of the stigma-reduction interventions in the intervention hospitals vs. control hospitals. In addition and similar to the analysis of the pre-intervention data, **SEM** – under guidance of the Flanders promoter – will allow us to test a path model which optimally reflects the complex interrelationships between our different key concepts and the HIV/AIDS and TB stigma in the healthcare setting.

All **qualitative data** will be entered into NVivo (qualitative data-management software) and two experienced analysts will conduct a thematic analysis – where text segments are assigned basic codes, are organized into categories, and then explored for relationships and overall themes. Transcripts will first be read and re-read to establish a preliminary coding structure. Two researchers will then independently code the scripts using the pre-determined codes, but also adding new, emerging codes. Coding will then be interrogated to establish inter-coder reliability, but also with a view to reaching conceptual alignment on existing and emerging codes. The goal of this in-depth analysis is to uncover the mechanisms (*why* and *how*) through which interventions have impacted (or failed to impact) stigma levels in the hospitals.

## Knowledge on the stigma-reduction intervention is disseminated

### Knowledge on stigma reduction is disseminated to different stakeholders

*Firstly*, we will share our findings with the international scientific community via **peer-reviewed publications** in journals with an SCI-impact factor (e.g*. Social Science & Medicine*, *Health Services Research*, *AIDS Care*, etc.). We will present the results at (inter)national conferences (*Union World Conference on Lung Health*, *International Conference on AIDS & STIs in Africa*, etc.). *Secondly*, we will share our findings with the **healthcare workforce** (e.g. hospital CEOs, HCWs, occupational health representatives) in a series of facility-level workshops. These workshops will aim to raise awareness around the problem of HIV and TB stigma in hospitals as well as suggest evidence-based strategies to combat it. *Finally*, we will present our results to the **FSDoH** in a study report that includes a list of evidence-based recommendations to combat HIV and TB stigma in South African health facilities. In this way we hope to contribute to the development and implementation of effective and sustainable stigma-reduction policies in a country heavily affected by this dual epidemic.

# Relevance of the Project

Because production of healthcare personnel has not kept pace with **(1)** population growth, **(2)** increasing care needs due for a large part to the TB-HIV co-epidemic, and **(3)** attrition of health professionals emigrating and leaving the public service , South Africa’s health system is confronted with a **severe human resource shortage**: for example, 4 of every 10 professional nurse posts in South Africa is currently vacant – causing the active healthcare workforce to be severely overburdened. Focusing on HIV/AIDS and TB, the co-epidemic lays a “*double burden*” on human resources: HCWs have to “*shoulder the impact of a rapidly increasing disease burden in the general population*”; in addition, they have to “*cope with increased morbidity and mortality in* [their] *own ranks*” [3].

It is therefore most important to optimally apply and support the limited available human resources for health. Major current South African health system reform initiatives are **National Health Insurance (NHI)** and **Primary Health Care Re-engineering**, both recognize this need and seek to strengthen health system effectiveness by way of better deployment and utilization of HCWs. The proposed project adheres to these broader policy reforms by finding evidence-based ways to improve working conditions of the healthcare workforce through minimizing HIV and TB stigma in the healthcare setting.

Regarding HIV/AIDS-TB policy, South Africa’s Minister of Health, Dr. Aaron Motsoaledi, recently (24/7/2014) announced his **HIV/AIDS 2030 plan** (including a large TB section). Stigma is acknowledged as a barrier to testing as well as to presenting for treatment. By concentrating efforts on reducing stigma among HCWs the proposed project links with and supports the aims of the 2030 plan, and furthermore, reaches a population group that plays an essential role in the fight against the HIV and TB co-epidemic in a high-burden context.

Action is thus urgently needed to optimally support the available human resources for health care. Our study – aiming to produce evidence-based interventions to reduce HIV/AIDS and TB stigma among HCWs – thus has **clear development relevance**. Decreased stigma is likely to result in a greater uptake of occupational health services and result in more convenient and timely testing and treatment for HCWs, potentially resulting in lower morbidity and mortality among scarce health personnel. Beyond the scope of the current project, it can, due to less time taken out of work to seek health services elsewhere, also lead to less absenteeism from work. In the long term, this can also produce a double positive impact on the quality of care for patients: **(1)** the lower attrition of health professionals due to illness could shorten waiting times and improve service delivery, and **(2)** the lower levels of stigma in the health facility should improve the quality of service to HIV/AIDS and TB patients in these facilities.

# Beneficiaries / target group

## Direct beneficiaries

The direct beneficiaries of this project are HCWs and selected intervention hospitals in the Free State:

- HCWs in intervention hospitals are expected to experience reduced HIV and TB stigma in the workplace.
- Healthcare workers should find that the environment in which they seek care has improved.
- Intervention hospitals are expected to benefit from developing a workplace that is better sensitised to human rights and responsibilities regarding the need to stop HIV and TB stigma

Following our Stigma Score Pilot Study in one hospital in the Free State (completed and with peer-reviewed publications in process), and following meetings with a range of key FSDoH stakeholders, we find excellent buy-in to the proposed study. Given a participatory approach to intervention design and delivery, and given that they are the targets as well as the vehicles of the interventions, FSDoH staff will play a central role in the research and particularly implementation of its intervention.

## Indirect beneficiaries

It is anticipated that HCWs who are better informed about and sensitised to HIV- and TB-stigma will be able to respond to and serve **patients** more humanely and effectively. However, this research is not designed to measure or evaluate the effect of the intervention *on patient populations* – as it specifically targets HCWs.

The proposed project will not only address regional social challenges but also deepen the academic collaboration between the partners: the project addresses the academic needs – namely (1) a shortage of quantitative methodology skills (through workshops) and (2) a shortage of qualified junior researchers (by including a Master’s scholarship) – of the South partner. The project will thus build **research capacity in the South** which can be usefully applied in future research efforts in this resource-limited setting.

# Assumptions, preconditions, sustainability

## Support from key stakeholders FSDoH and facility staff

The full support from the FSDoH and the full cooperation of the staff at the different experimental and control hospitals will be integral to the successful execution of the project. As mentioned above, the Stigma Score Pilot Study and subsequent meetings with a range of key FSDoH stakeholders and hospital representatives has resulted in the full support of these stakeholders to the proposed study. Given the participatory approach to intervention design and delivery, the research team is confident that these stakeholders will continue to take up their central role in the research and its implementation.

## Ethical clearance

The study is subject to the approval of the Ethics Committee in the Faculty of Health Sciences at the UFS and UA Ethical Clearance Committees. To acquire this approval, the study will be executed in accordance with the 2002 version of the Declaration of Helsinki (informed consent, full confidentiality, etc.).

## Assumptions underlying the programme logic

Following a series of stigma-reduction interventions over time, which are strongly complemented by ongoing support and mentoring of occupational health nurses—or staff nurses/doctors providing HIV and TB services to other healthcare workers in their facilities—to initiate and provide ART in occupational health clinics, HIV and TB-related stigma in the workplace will diminish; this will lead to greater willingness among HCWs to access available HIV & TB testing and treatment. .

# Project sustainability

## Sustainability of the developmental benefits

The sustainability of the health system strengthening benefits will be ensured in two ways. *Firstly* and on a *policy level*: at the discretion of the FSDoH, the research will involve staff in a division within the FSDoH — the Employee Assistance Programme (EAP) — mandated to enhance the welfare of HCWs. Knowledge transfer within this formal division, supporting the efforts and development of champions, and active recruitment of new champions will assist in building **longer term continuity**.

## Sustainability of the academic benefits

Antwerp University and the CHSR&D already have a strong working relationship. The proposed project is the next – necessary – step to ensure the continuity of the ongoing collaboration: it will build the urgently required new research capacity at the local partner. The workshops in research methodology and the training of a locally-based Master’s student are tangible steps taken by the current project to ensure the sustainability of the academic benefits generated by the project. The appointment of the North PI as Research Associate of the CHSR&D further exemplifies the long-term commitment of both partners. The North-South team will continue to seek opportunities to continue the ongoing exchange of research expertise, and sharing of subject-based knowledge.

# References

**[1]** UNAIDS, *Global report* *on the global AIDS epidemic*, 2013, Joint United Nations Programme on HIV/AIDS. p. 198.

**[2]** WHO, *Global Tuberculosis Report 2013*, 2013, World Health Organization. p. 306.

**[3]** Department of Health, *Annual Performance Plan 2012/13-2014/15*, 2012, Republic of South Africa. p. 100.

**[4]** Joshi, R., et al., Tuberculosis among Health-Care Workers in Low- and Middle-Income Countries: A Systematic Review. *PLoS Med*, 2006. 3(12): p. e494.

**[5]** URC South Africa, *Tuberculosis in Healthcare Workers: Findings from South Africa*, 2013, University Research Co. LLC & Desmond Tutu Tuberculsosis Centre. p. 2.

**[6]** Adamsi, S., et al., Occupational Health Challenges Facing the Department of Health: Protecting employees against tuberculosis and caring for former mineworkers with occupational health disease, in *South African Health Review 2012/13*, A. Padarath and R. English, Editors. 2013, Health Systems Trust: Durban. p. 67-82.

**[7]** Ncayiyana, D., Doctors and nurses with HIV and AIDS in sub-Saharan Africa. *BMJ*, 2004. 11(329(7466)): p. 584-585.

**[8]** Feeley, F.G., et al., A Successful Workplace Program for Voluntary Counseling and Testing and Treatment of HIV/AIDS at Heineken, Rwanda. *International Journal of Occupational and Environmental Health*, 2007. 13(1): p. 99-106.

**[9]** Corbett, E.L., et al., Tuberculosis in sub-Saharan Africa: opportunities, challenges, and change in the era of antiretroviral treatment. *The Lancet*, 2006. 367(9514): p. 926-937.

**[10]** WHO, ILO, and UNAIDS, *The joint WHO-ILO-UNAIDS policy guidelines on improving health workers’ access to HIV and TB prevention, treatment, care and support services 2010*: Geneva.

**[11]** Nyblade, L., et al., Combating HIV stigma in health care settings: what works? *Journal of the International AIDS Society*, 2009. 12(1): p. 15.

**[12]** Uys, L., et al., Evaluation of a Health Setting-Based Stigma Intervention in Five African Countries. *AIDS Patient Care & STDs*, 2009. 23(12): p. 1059-1066.

**[13]** Holzemer, W.L., et al. Measuring HIV stigma for PLHAs and nurses over time in five African countries. *SAHARA-J* 2009. 6, 76-82.

**[14]** Chirwa, M.L., et al., HIV Stigma and Nurse Job Satisfaction in Five African Countries. *Journal of the Association of Nurses in AIDS Care*, 2009. 20(1): p. 14-21.

**[15]** Varas-Díaz, N. and T.B. Neilands, Development and validation of a culturally appropriate HIV/AIDS Stigma Scale for Puerto Rican health professionals in training. *AIDS Care*, 2009. 21(10): p. 1259-1270.

**[16]** Dlamini, P.S., et al., Verbal and Physical Abuse and Neglect as Manifestations of HIV/AIDS Stigma in Five African Countries. *Public Health Nursing*, 2007. 24(5): p. 389-399.

**[17]** Mbonu, N.C., B. van den Borne, and N. De Vries, Stigma of People with HIV/AIDS in Sub-Saharan Africa: A Literature Review. *Journal of Tropical Medicine*, 2009.

**[18]** Greenhalgh, T., et al., *Diffusion of Innovations in Health Service Organisations. A systematic literature review* 2008: Blackwell Publishing;
