# Supplementary material for: Towards a health-enabling working environment - developing and testing interventions to decrease HIV and TB stigma among healthcare workers in the Free State, South Africa: study protocol for a randomised controlled trial
Source: Trials. 2018 Jul 4;19:351. doi: 10.1186/s13063-018-2713-5 (PMC6031140; doi:10.1186/s13063-018-2713-5)
Supplement: Supplementary file 7 — FSDoH Permission Baseline phase 2015. (DOCX 83 kb) [file 13063_2018_2713_MOESM7_ESM.docx]

Dr. A Rau

Centre for Health Systems research and development

UFS

Bloemfontein

## 28 July 2015

**ea.th**

Depa rtment of Hea lth

## FREE STATE PROVINCE

Dear Dr A Rau

Subject: Towards a health enabling working environment: Developing and testing intervention to decrease HIV and

TB sti ma amon	health care workers in the Free State South Africa.

- Pennission is hereby granted for the above - mentioned research on the following conditions:
- Participation in the study must be voluntary.
- A written consent by each participants must be obtained.

# •

•

•

•

•

•

•

•

•

•

•

•

•

Trus

Serious adverse events to be reported and/or tennination of the study.

Ascerta in that your data collection exercise neither interferes with the day to day running of the facilities nor the perfonnance of duties by the respondents or health care workers.

Confidentiality of infonnation will be ensured and no names will be used.

Research results and a complete report should be made available to the Free State Department of Health on completion of the study (a hard copy plus a soft copy).

Progress report must be presented not later than one year after approval of the project to the Ethics Committee of the University of the Free State and to Free State Department of Health.

Any amendments, extension or other modification s to the protocol or investigators must be submitted to the Ethics Committee of the University of the Free State and to Free State Department of Health.

Conditions stated in your Ethical Approval letter should be adhered to and a final copy of the Ethics Clearance Certificate should be submitted to khusem j (a; fshcalth. gov.za or scbcclats(ci..fshcalth.gov.za before you commence with the study

No financial l iability will be placed on the Free State Department of Health

Please discuss your study with the institution managers/CEOs on commencemen t for logistical arrangements Department of Health to be fully indemnified from any harm that participants and staff experiences in the study

Researchers will be required to enter in to a fonnal agreement with the Free State department of health regulating and fonnalizing the research relation ship (document will follow)

You are encouraged to present your study findings/results at the Free State Provincial health research day Future research will only be granted pennission if correct procedures are followed see [http://nhrd](http://nhrd/).hst.org.za

order.

## HEAD: H

Date:	.2-0]1

[www.fs.gov.za](http://www.fs.gov.za/)
