# Supplementary material for: Towards a health-enabling working environment - developing and testing interventions to decrease HIV and TB stigma among healthcare workers in the Free State, South Africa: study protocol for a randomised controlled trial
Source: Trials. 2018 Jul 4;19:351. doi: 10.1186/s13063-018-2713-5 (PMC6031140; doi:10.1186/s13063-018-2713-5)
Supplement: Supplementary file 8 — FSDoH Permission Intervention phase 2017. (DOCX 47 kb) [file 13063_2018_2713_MOESM8_ESM.docx]

**health**

Depa rtm en t of H ea l t h

## FREE STATE PROVINCE

Dr. A Rau

Centre for llealth S)stems research and development

tii:s

Blocmfonlein

## 13 Februa ry 2017

Dear Dr A Rau

Subject: Towards a health enabling working environment: Developing and testing intervention to decrease HIV and TB stigma

a mon	hea lth care workers in the Free State South Africa.

- Permission is hereby granted for the above mentioned research on the follov..ing cond itions:
- This letter replaces that letter dated 28 July 2015 and should be considered the only approval letter.
- Participation in the study must be vol untary .
- A written consent by each participant s must be obtained.
- Serious adverse events to be reported and/or termination of the study.
- Ascertain that your data collection exercise neither interferes with the day to day running of the facilities nor the performance of duties by the respondents or health care workers.
- Confidentiality of information \\ ill be ensured and no names will be used.
- Research results and a complete report should be made available to the Free State Department of I leaIth on completion of the study (a hard copy plus a soft copy).
- Progress report must be presented not later than one year after approval of the project to the Ethics Committee of the University of the Free State and to Free State Department of I lealth.
- Any amendments, extension or other modifications to the protocol or investigators must be submitted to the Ethics Committee of the University of the Free State and to Free State Department of llealth.
- Conditions stated in your Ethical Approval letter should be adhered to and a final copy of the Ethics Clearance Certificate should

be submitted to seheclats *a* fshealt h.gm *.1a* before you commence with the study

- No financial liability \\ill be placed on the Free State Department of Health
- Please discuss your study with the institution managers/CEOs on commencement for logistical arrangements
- Department of I leaIth to be fully indemnified from any harm that participants and staff experiences in the study
- Researchers will be required to comply with the MOU.
- You are encouraged to present your study findings/results at the Free State Provincial health research day
- F	re research will only be granted permission if correct procedures are followed see h1tp:/1nhn.l .hs1.org.1a ve in order.

Dr D Motau

# HEAD:HEALT1

Date:	z.f Joz t -T

Head :Health

PO Box 227. Bloemfotein,9300

41t1 Floor,Executive Suite, Bophelo House,cnr Maitland and,Harvey Road, Bloemfotein

Tel (051) 4081646 Fax (051) 408 1556 e-mall:khusem1@fshealth gov za@fshealth .gov z;l/cllikobvu.J.>@fshealt.!Lg

www.fs gov za
